# Structuring evolution: biochemical networks and metabolic diversification in birds

**DOI:** 10.1186/s12862-016-0731-z

**Published:** 2016-08-25

**Authors:** Erin S. Morrison, Alexander V. Badyaev

**Affiliations:** Department of Ecology and Evolutionary Biology, University of Arizona, Tucson, AZ USA

**Keywords:** Network structure, Metabolic pathways, Phenotypic diversity

## Abstract

**Background:**

Recurrence and predictability of evolution are thought to reflect the correspondence between genomic and phenotypic dimensions of organisms, and the connectivity in deterministic networks within these dimensions. Direct examination of the correspondence between opportunities for diversification imbedded in such networks and realized diversity is illuminating, but is empirically challenging because both the deterministic networks and phenotypic diversity are modified in the course of evolution. Here we overcome this problem by directly comparing the structure of a “global” carotenoid network – comprising of all known enzymatic reactions among naturally occurring carotenoids – with the patterns of evolutionary diversification in carotenoid-producing metabolic networks utilized by birds.

**Results:**

We found that phenotypic diversification in carotenoid networks across 250 species was closely associated with enzymatic connectivity of the underlying biochemical network – compounds with greater connectivity occurred the most frequently across species and were the hotspots of metabolic pathway diversification. In contrast, we found no evidence for diversification along the metabolic pathways, corroborating findings that the utilization of the global carotenoid network was not strongly influenced by history in avian evolution.

**Conclusions:**

The finding that the diversification in species-specific carotenoid networks is qualitatively predictable from the connectivity of the underlying enzymatic network points to significant structural determinism in phenotypic evolution.

**Electronic supplementary material:**

The online version of this article (doi:10.1186/s12862-016-0731-z) contains supplementary material, which is available to authorized users.

## Background

Only a small proportion of theoretically possible changes seemed to be realized in phenotypic evolution and diversification, with some outcomes appearing recurrently whereas others are seemingly forbidden [1–5]. Such determinism and predictability of phenotypic outcomes is surprising considering the dimensionality of the genome, the proteome, and the developmental dynamics linking them and point to the existence of constraints in phenotypic variation. Theoretical and empirical studies have suggested that such constraints may be a reflection of the connectivity of the network of interactions among elements such as genes, proteins, enzymes and metabolites (defined here as a deterministic network) caused by genomic or developmental epistasis [1, 6–11], internal integration during development [12–15], and physical stability or historical contingency of gene and protein associations [16–22]. Direct examination of the correspondence between opportunities for diversification imbedded in such networks and realized phenotypic diversity is needed to illuminate the structural properties of networks that delineate phenotypic diversity.

**Fig. 1:**
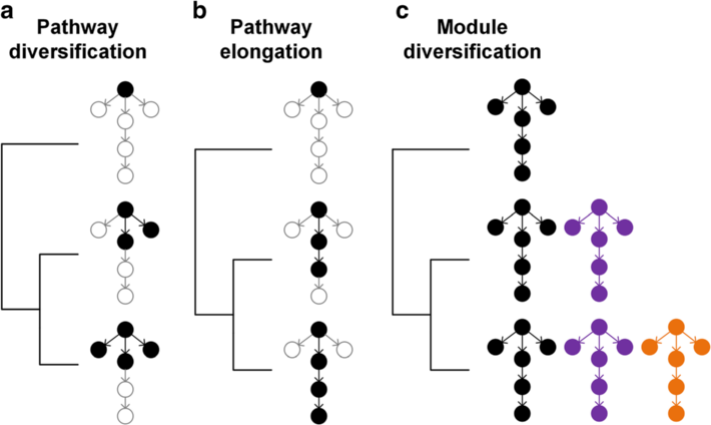
The structure of a deterministic network and potential evolutionary trajectories. The possible interactions (*arrows*) between elements (*small circles*) represent potential opportunities for diversification on a deterministic network (shown in *grey*). The *black*, *purple* and *orange* shaded portions of the network show examples of different expressed networks, with each color denoting a different functional module made up of different elements and interactions. (**a**) Under the pathway diversification scenario, elements with the most interactions (higher connectivity) should be most conserved across networks, and the number and identity of the interactions associated with these connected elements should differ across networks. (**b**) Under the pathway elongation scenario, elements at the beginning of a sequential pathway of reactions should be the most conserved across networks, and the pathway length (the number of reactions that separate one element from another) and elements located further away from the start of the pathway should differ between networks. (**c**) Under the module diversification scenario, differences between networks are the result of the gain or loss of entire modules (unique groups of functionally coupled elements and interactions) and the gain or loss of elements would not be related to their connectivity or to their distance from a starting element in a pathway

Phenotypic diversification on a deterministic network is the result of the gain or loss of elements and interactions that convey different fitness [1, 3, 22]. Mechanistically, the evolutionary representation and variability of network elements tends to be associated with their topological positions [23–28]. In particular, two structural properties of networks – the number of reactions per element, which represents the connectivity of the network, and the number of reactions that separate elements in a network, which defines the length of pathways between elements in the network – provide distinct ways by which elements and interactions in the network are gained or lost and result in different patterns of phenotypic diversification (Fig. 1) [29–33].

Greater connectivity of an element – the number of direct interactions it has with other elements in a network – enables an evolving lineage to include different elements that both directly interact with the same element [34–36]. In this mode of network diversification (hereafter pathway diversification), the gain of different interactions associated with the same element represents the start of divergent pathways comprised of unique elements and interactions (Fig. 1a). For example, in metabolic networks, the use of different enzymatic reactions from the same substrate metabolite produces different products resulting in distinct metabolic pathways. Theory and empirical data suggest that metabolic and protein networks commonly evolve by the preferential attachment of new enzymatic reactions or protein interactions to the most connected elements in these networks [24, 34, 37, 38]. Correspondingly, the genes underlying proteins and enzymes with greater connectivity tend to be represented in a greater number of taxa, have longer evolutionary persistence and lower rates of evolutionary change than elements with fewer direct interactions in a network [23, 39, 40]. Thus, the divergence among species’ networks should be driven by the gain or loss of interactions among highly connected elements, whereas the connected elements themselves should be conserved across species. Differences in the number of interactions that start from these conserved elements should be reflected in differences in the overall network connectivity (number of interactions per element) across species’ networks, because a greater number of opportunities exist for species to express different interactions at densely connected compounds. If pathway diversification causes divergence among species’ networks, then we expect differences in the elements and interactions present across species networks to increase with the differences in the connectivity of their networks, such that interactions and elements associated with the most connected compounds in the network should vary the most across species.

The length of pathways – the number of interactions (e.g., enzymatic reactions) that connect elements in a network – enables an evolving lineage to express different elements and reactions along *the same* pathway. This mode of network diversification (hereafter pathway elongation), results from differences in the number of sequential interactions from the same starting element (Fig. 1b). Most genes, proteins, and metabolites are regulated by multistep interactions [35, 41] and thus in most cases, the activation or expression of an element is dependent on several prior interactions. Changes in interactions at the beginning of a pathway may prevent the expression of interactions located further downstream in the pathway and result in shorter pathways and the loss of elements. Alternatively, the addition of a new interaction to the end of a pathway can increase the length of the pathway and produce a novel product. Models of network growth and empirical results suggest that most of the change in networks occurs at their periphery, such that terminal elements are most likely to be gained or lost, whereas the central or upstream elements are the most conserved [39, 42–44]. Longer pathways between elements in a network therefore provide more opportunities for the use of different numbers of sequential reactions from the same starting element, such that some species networks only express the intermediate elements that lie along a pathway of interactions from one element to another and the final product is never expressed. If network diversification is driven by differences in the elongation of a sequence of interactions among species, then we expect species’ networks to have different pathway lengths from the same starting element. The difference in the length of the pathways among species’ networks should be reflected in the diversification among the elements and interactions present in each species. In this case, the elements located at the beginning of pathways should be conserved across networks, and species’ networks should diverge more from each other at elements located closer to the ends of potential pathways.

Networks are often organized in discrete functional modules in which a group of metabolites, enzymes, genes, or proteins interact more often with each other than with other elements in the network [45, 46]. Functional modules play an important role in the evolvability of organisms [47–51]. Empirical studies have shown that genes in the same regulatory modules tend to be co-expressed [52–55], resulting in similar evolutionary rates of proteins in the same modules [56, 57]. Additionally, genes that underlie within-module enzymatic reactions have similar rates of evolutionary gain and loss (e.g., [58, 59]), such that multiple enzymatic reactions that comprise a pathway are gained or lost together. Therefore, another mode of network divergence among species could be the result of the gain or loss of complete functional modules (hereafter module diversification) (Fig. 1c). If this is the case, then species should differ in modules they express, and neither the connectivity of elements nor the length of a pathway between elements in a network should be related to the differences in species’ networks.

Here we examined the extent to which the structure of enzymatic reactions in the global carotenoid network – that comprises all of the documented enzymatic reactions among naturally occurring carotenoids (Additional file 1a) – is associated with patterns of avian diversification in carotenoid-producing metabolic networks. The connectivity and topology of enzymatic reactions of the global carotenoid network have evolved largely in the context of bacterial evolution (e.g., [60, 61]) and subsets of this global network are utilized in the carotenoid metabolism of all lineages studied to date, such as fungi, plants, insects and animals (e.g., [62, 63]). Here we studied the patterns of utilization of this network associated with the production of carotenoid pigmentation in the plumage and integument of 250 bird species. Specifically, we were interested in the effect of the structure of the global metabolic network on the frequency of occurrence of individual carotenoid compounds and reactions across species.

**Fig. 2:**
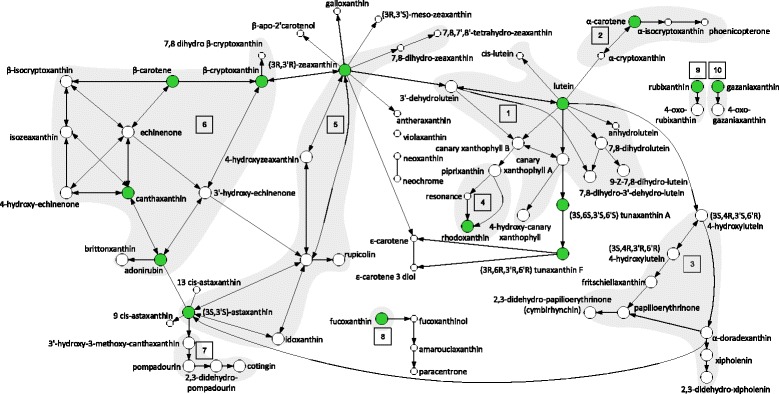
Schematics of the connected global enzymatic network of carotenoid compounds (66 compounds, 97 enzymatic reactions) found in species under this study (Additional files 1 and 2). *Green* nodes show dietary carotenoids. The distinct shaded areas represent the module assignments for the 53 compounds expressed at least once across species’ networks using simulated annealing [71, 72]. The numbers in the *squares* for each module denote the module number that corresponds to the module assignments for each compound in Additional file 1c

**Fig. 3:**
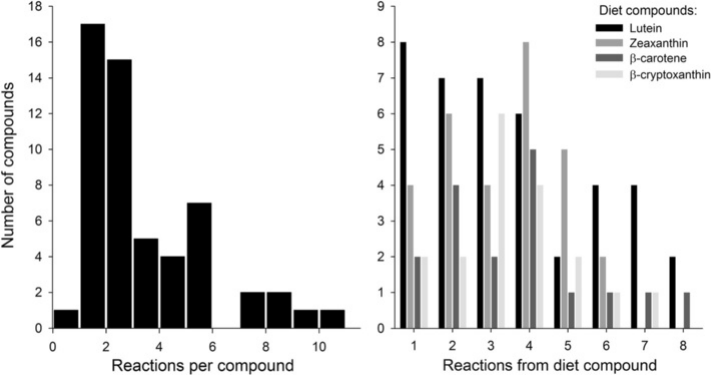
Structural diversity of carotenoid compounds in the avian space of the global carotenoid metabolic network (Fig. 2). Compounds differ in connectivity (reactions per compound), shown in the histogram on the *left*, and their distance (number of reactions) from the four main dietary (starting) compounds (lutein, zeaxanthin, β-carotene, β-cryptoxanthin), shown in the graph on the *right*

In birds, metabolism of carotenoids expressed in feathers and integument necessarily starts with the consumption of dietary carotenoids (e.g., [64, 65]). This property of avian carotenoid biosynthesis allows for the identification of the starting points of metabolic pathways in species’ networks and provides an opportunity to distinguish the effects of pathway diversification from the effects of pathway elongation and module diversification on network divergence across species. In birds, pathway diversification from the same highly connected compounds, pathway elongation starting at the same dietary compounds, or the consumption of different dietary compounds representing different functional modules in the network could produce evolutionary transitions across species’ networks. In the global carotenoid network, opportunities for pathway diversification and elongation vary across metabolic pathways that start at different dietary carotenoids (Figs. 2 and 3). Additionally, the consumption of different dietary compounds results in access to different enzymatic reactions and metabolites that could comprise different functional modules (Fig. 2). Here, we first mapped species’ carotenoid networks onto the global avian carotenoid metabolic network [66] and examined whether differences in enzyme connectivity or relative pathway position of individual carotenoid compounds were associated with their evolutionary representation among species. We then repeated these analyses for biochemical modules of interconnected elements and examined their evolutionary representation in relation to their structural properties. We examined the relative contribution of enzymatic connectivity, metabolic pathway lengths, and module representation on network divergence and identified the structural properties of both individual compounds and modules associated with diversification hotspots on the global carotenoid network. We discuss the extent to which the structure of the carotenoid metabolic network can be used to understand and predict patterns of realized phenotypic diversity.

## Methods

### Data collection and metabolic network construction

The global carotenoid biosynthesis network includes all of the enzymatic reactions that occur among naturally-occurring carotenoids in bacteria, plants, fungi and animals (Additional file 1a, [66]). This network delineates biochemical pathways of carotenoid biosynthesis based on the chemical properties of the compounds. We collected an exhaustive list of all the carotenoid compounds and reactions documented in birds (*n* = 339 species), using carotenoids that are found in plumage, integument (bill, tarsi, skin), plasma, liver, fat, feces, retina, and seminal fluid, or are known to be consumed in the diet (Additional file 1b; data current as of July 2015). The chromatography and mass spectrometry methods that are listed in Additional file 1b document the presence or absence of specific compounds against known standards. All of the distinct compounds identified in the species of birds were then used to construct the “avian subset” of the global carotenoid metabolic network, consisting of 66 carotenoids and 97 enzymatic reactions (Fig. 2). The global metabolic network was then used as a template to construct 250 species-specific carotenoid metabolic networks between known dietary carotenoid compounds (the upstream elements of carotenoid metabolic networks in birds), metabolized compounds (e.g., circulating in plasma or found in other organism tissues), and the expressed compounds identified from species’ plumage and integument (Additional file 2). Briefly, after mapping compounds found in the diet, plasma, and plumage or integument of species under this study on the “avian space” of the global carotenoid biosynthesis network (Fig. 2), we recorded biochemical pathways that link dietary, intermediate and plumage-expressed compounds for each species (Additional files 1b and 2; details and justification in Badyaev et al. [66], which also see for phylogenetic analyses of avian carotenoid networks). For species that had no known dietary or intermediate compounds (but not both), missing compounds and reactions were assigned based on the mapping of the species’ known compounds and reactions on the global network and recording all biochemically possible connections (e.g., between a known dietary and a known expressed compound or between a known intermediate and a known expressed compound and a possible dietary compound). Networks were not built for species if the carotenoids expressed in their plumage or integument were unknown even when all other components of the network were documented. Thus, not all of the compounds and reactions in the avian subset of the global carotenoid metabolic network (Additional file 1a, Fig. 2) were present in the species-specific networks. In the 250 species-specific complete networks that were constructed, 53 compounds and 81 enzymatic reactions occurred at least once. Species under this study represent eleven avian orders (Anseriformes, Charadriiformes, Ciconiiformes, Columbiformes, Galliformes, Passeriformes, Pelecaniformes, Phaethontifromes, Phoenicopteriformes, Piciformes, Trogoniformes) and span over 110 MYA of avian carotenoid diversification (Fig. 4a, 4b, 4c, 4d and 4e, Additional file 3) [66].

**Fig. 4:**
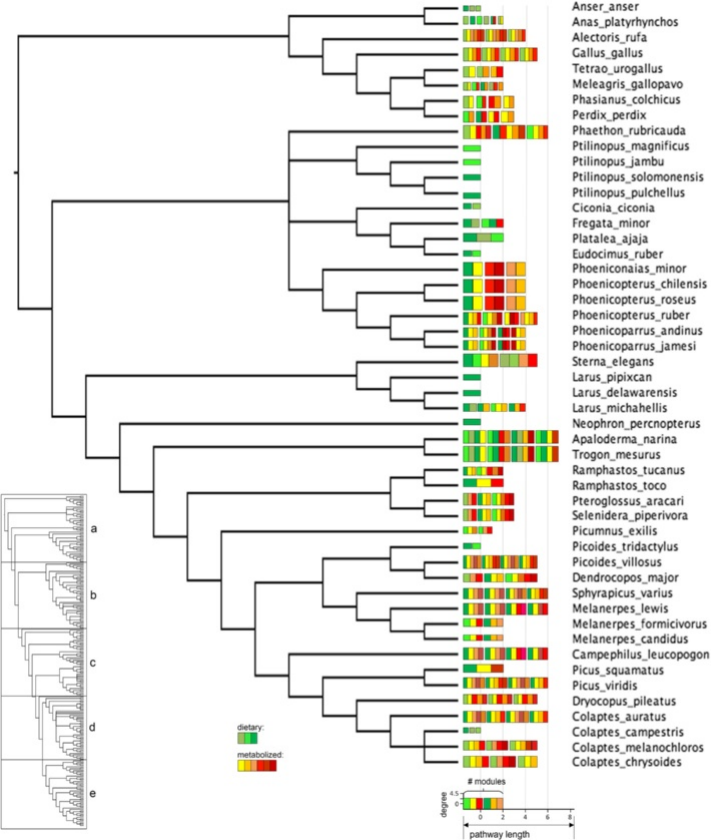
(a) Consensus tree of the non-passerine species in this study showing, for each species’ metabolic network, the number of compounds (number of *bars*; *green bars* –distinct dietary carotenoids; *yellow*, *orange* and *red bars* – metabolically derived compounds), average degree (*y*-axis of the legend), number of modules (number of *bar* groups), pathway length (*x* –axis of the legend, number of enzymatic reactions from the closest dietary compound). The tree is a part of a majority rule consensus tree of 249 species based on 1,000 randomly sampled trees from the Hackett All Species pseudo posterior distribution from Jetz et al. [116] (Additional file 3). The other subsets of the tree, show in the inset in the lower left corner, are displayed in Figures 4b, 4c, 4d, and 4e

**Fig. 4:**
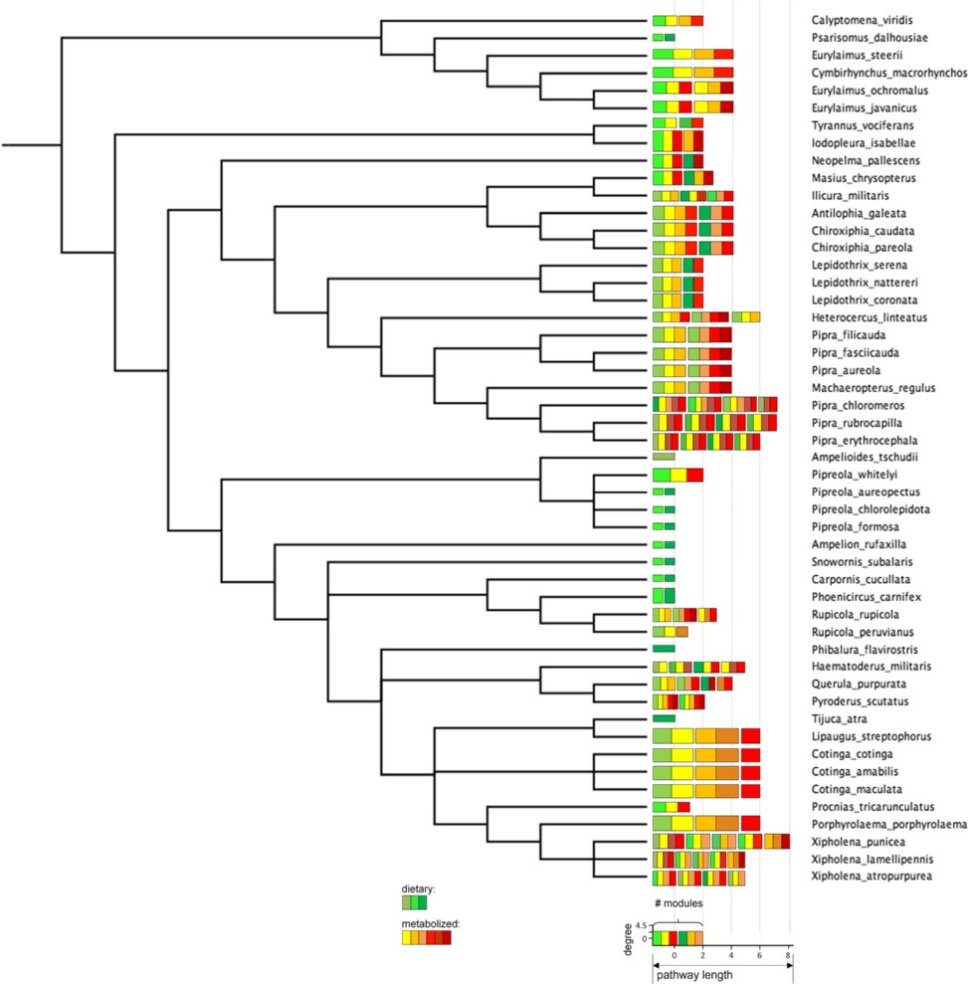
(b) Consensus tree of the suboscine species under this study. Legend in Figure 4a

**Fig. 4:**
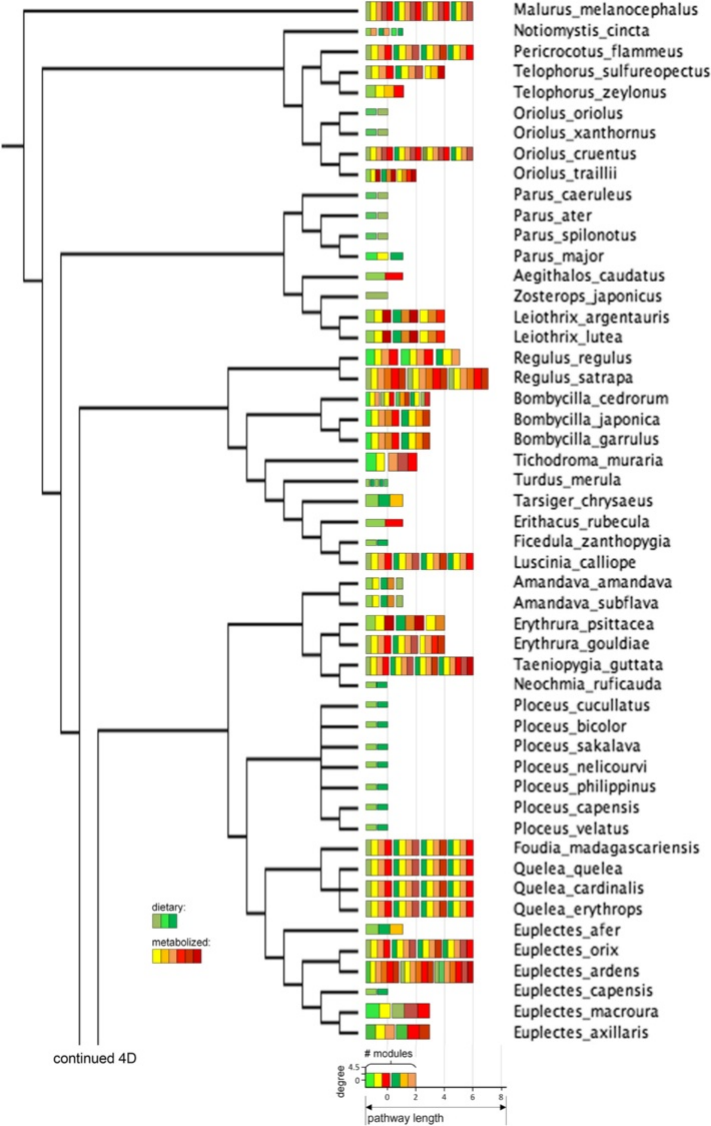
(c) Consensus tree of a subset the oscine species under this study. Legend in Figure 4a

**Fig. 4:**
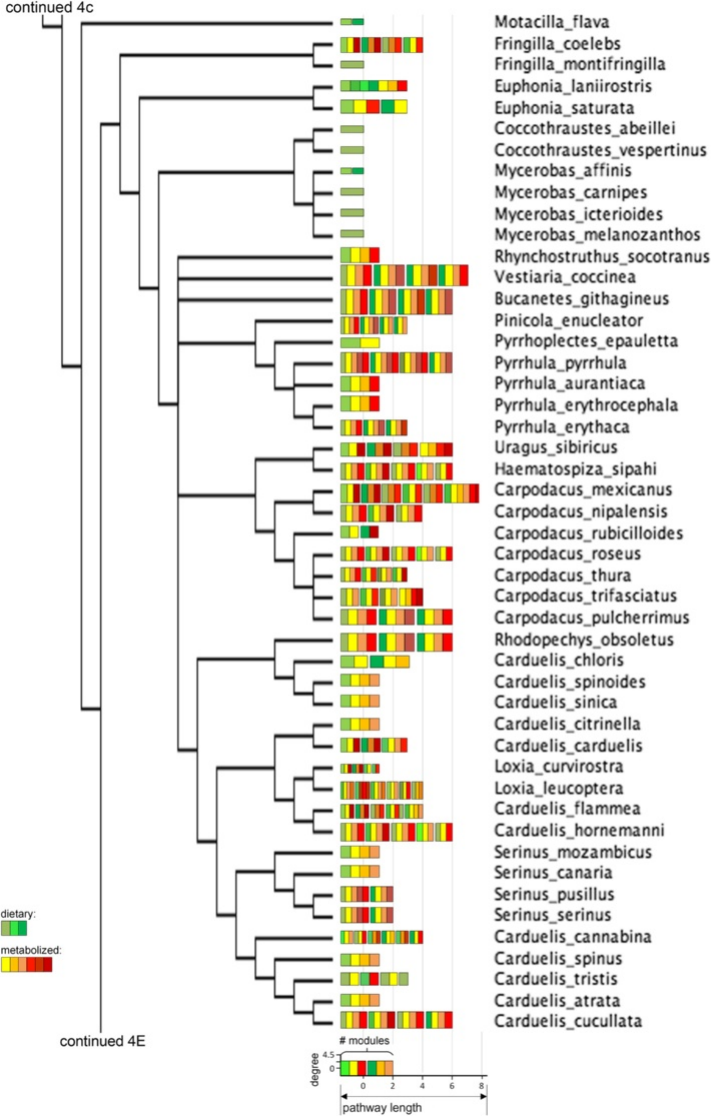
(d) Consensus tree of a subset of the oscine species under this study. Legend in Figure 4a

**Fig. 4:**
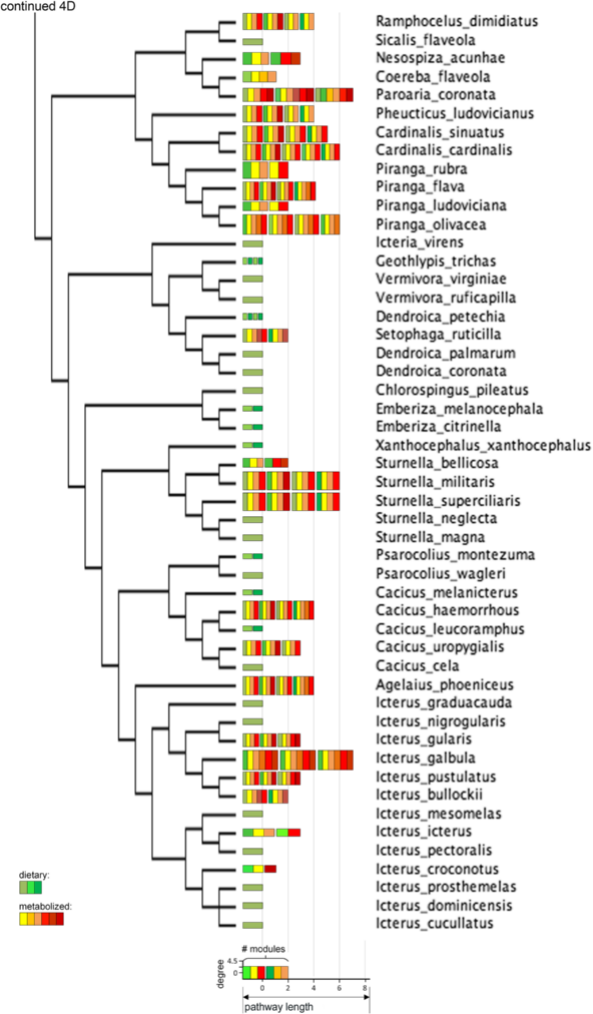
(e) Consensus tree of a subset the oscine species under this study. Legend in Figure 4a

### Metabolic distance and modularity in networks

We used a modified metabolic distance based on the Jaccard distance [67] and Rodrigues and Wagner [68] to calculate the fraction of reactions and compounds differing between any two metabolic networks. Species’ networks were coded based on the presence of compounds and reactions in the avian subset of the global carotenoid metabolic network. The uncorrected *P*-distance is the fraction of the number of compounds and reactions that differ between each pair of networks (*d*) out of the total number of compounds and reactions in the global network (*N*_G_):

$$ P\kern0.5em =\kern0.5em \frac{d}{N_G} $$

The pairwise *P*-distances were computed in Mesquite (version 3.03) [69] using the PDAP:PDTREE (version 1.16) package [70]. The metabolic distance (*D*) between networks represents the fraction of compounds and reactions in which two networks differ out of the total number of compounds and reactions that occur in each of the networks:

$$ D\kern0.5em =\kern0.5em \frac{d}{N_1\kern0.5em +\kern0.5em {N}_2} $$

where *N*_1_ and *N*_2_ are the total number of compounds and reactions in networks *S*_1_ and *S*_2_, respectively. The 53 compounds expressed in the global carotenoid network at least once among the species’ networks were partitioned into ten structurally defined modules based on the density of the compounds’ enzymatic interconnectivity using the simulated annealing program *netcarto* (https://amaral.northwestern.edu/resources/software/netcarto) [71, 72]. This approach to module partitioning has previously been used to reliably assign metabolites to the correct functional pathway based only on the structural properties of the metabolites [71]. In the avian carotenoid metabolic network, the modules are partitioned by different dietary compounds; seven of the ten modules include at least one starting, upstream dietary compound. For module assignments of the individual compounds in the global carotenoid metabolic network refer to Fig. 2 and Additional file 1c.

### Network structural measurements

For each compound in the avian carotenoid network (Fig. 2) we calculated the number of directly linked enzymatic reactions [73] and the distance from a dietary compound (minimum number of reactions between a compound and any of the dietary compounds in the network) to represent the connectivity and the pathway position of each compound, respectively. The connectivity (*C*) of each of the modules in the global network and each of the species’ networks was the average number of reactions per compound:

$$ C\kern0.5em =\kern0.5em \frac{r}{n} $$

where *r* is the total number of reactions in the module or network and *n* is the total number of compounds in the module or network. The diameter of each of the species’ networks is the shortest distance (number of reactions) between the two most distant dietary and expressed compounds in the network. The diameter of each of the modules in the global network is the fewest number of reactions between the two most distant compounds in the module. Both the connectivity of the species' networks and the modules and the diameter of the modules were computed using Cytoscape 2.8.2 [74] with NetworkAnalyzer 2.7 [75, 76] and RandomNetworks 1.0 [77].

### Species representation and realized phenotypic diversification

The species representation of a compound or reaction is the number of species that have this compound or reaction (e.g., [39]). Whereas species representation characterizes the evolutionary representation of a compound, it does not include information on species’ phylogenetic relationships, and instead enables the examination of metabolic network evolution from a structural, rather than historical perspective (e.g., [39]). In a companion study we found that the phylogenetic relationships among the species in this study were not reflected in the similarity of their biochemical networks; the small biochemical space on which birds diversify and the structure of the biochemical network instead leads to recurrent convergence of distantly related and ecologically distinct taxa in metabolic networks [66]. Having examined the historical sequence of exploration of the global carotenoid network by extant avian species in that study, here we explore whether the structure of the global carotenoid network is reflected in the pattern of network exploration across avian lineages. Several other studies have taken similar approaches to compare structural features of metabolic networks across species of bacteria, eukaryotes, and archaea independently of their phylogenetic relationships (e.g., [24, 35, 78]).

The realized diversification (*R*) of an enzymatic reaction was measured as the fraction of species that do not have a reaction among all of the species that have the substrate compound for the reaction (*n*_*c*_), where *n*_*r*_ is the number of species that have the reaction:

$$ R\kern0.5em =\kern0.5em \frac{n_c\kern0.5em -\kern0.5em {n}_r}{n_c} $$

An enzymatic reaction with a realized diversification score of zero represents a location in the network with little or no divergence between species’ networks along that part of a pathway; meaning that the enzyme is conserved across species that also have the enzyme’s substrate compound. The realized diversification of an enzymatic reaction with a score close to 1 represents a point of major divergence between species (i.e., the enzyme is only present in a small fraction of the total species that have the enzyme’s substrate compound).

## Results

### Global carotenoid network structural properties and diversity of species’ networks

Connectivity and the distance from dietary carotenoids of compounds varied widely in the avian subset of the global carotenoid network (Figs. 2 and 3). All but one compound were associated with at least one reaction to a maximum of 10 reactions. Non-dietary compounds were one to eight reactions away from starting dietary carotenoids (Fig. 3). The species’ networks (Fig. 4a, 4b, 4c, 4d and 4e; Additional file 1b) differed widely in the number of total compounds (1-21), number of reactions (0-46), connectivity (0-4.53 average reactions per compound), diameter length (0-8 reactions), number of modules (1-6), and number of dietary carotenoids (1-6).

### Structural determinants of compound occurrence among species

**Fig. 5:**
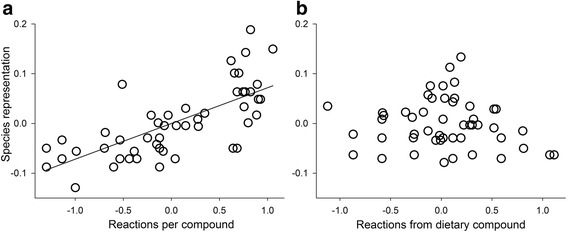
A compound’s connectivity contributed more to the compound’s occurrence than did the compound’s relative distance from a dietary compound. Shown are partial regressions of a compound’s species representation on (**a**), the number of reactions per compound and (**b**), its distance from a dietary compound

The connectivity of a compound contributed the most to its species representation; carotenoids with higher connectivity had greater species representation (Fig. 5a; *b*_*ST*_ = 0.73, *t* = 7.63, *P* < 0.001, *n* = 55). Species representation of a compound did not vary with its distance from a dietary carotenoid (Fig. 5b; *b*_*ST*_ = -0.07, *t* = -0.72, *P =* 0.48, *n* = 55).

### The role of modules in compound occurrence among species

**Fig. 6:**
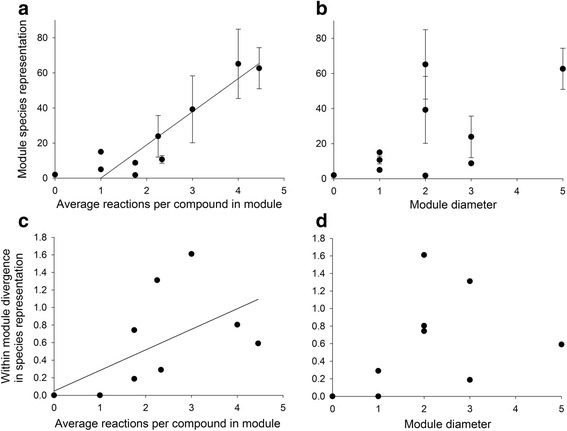
Species representations of interconnected compounds within modules were related to the connectivity, but not the length of pathways of these modules. Compounds in modules characterized by (**a**), greater overall connectivity were overrepresented across species’ networks, whereas the occurrence of compounds in modules was not related to (**b**), the diameter of the module. Vertical bars represent the standard error. Differences in the species representation of compounds in the same module increased with (**c**), greater module enzymatic connectivity, but was not related to (**d**), the diameter of the module

The representation of functional modules of the avian carotenoid network varied across species' networks (Fig. 6a and b). Modules of higher connectivity occurred in more species (Fig. 6a; Spearman’s *ρ* = 0.80, *P* = 0.006, *n* = 10), but the diameter of a module was not related to the occurrence of the module across species (Fig. 6b; *ρ* = 0.49, *P* = 0.15, *n* = 10). Differences in the numbers of species with each of the compounds in a module were correlated with the connectivity of the module (Fig. 6c; *ρ* = 0.74, *P* = 0.01, *n* = 10), but not with the diameter of the module (Fig. 6d; *ρ* = 0.59, *P* = 0.07, *n* = 10).

### Structural determinants of metabolic distance among species networks

**Fig. 7:**
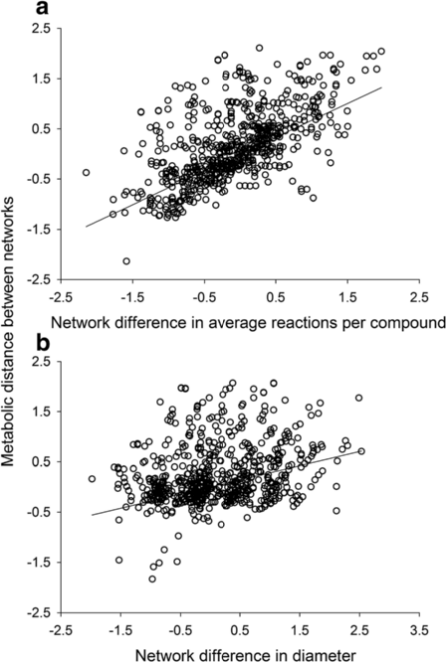
Differences in enzymatic connectivity contributed more to network divergence than differences in diameter. Shown are partial regression plots of the metabolic distance between pairs of species’ networks that share the same dietary (starting) compounds and the difference in (**a**), network connectivity and (**b**), diameter length between each pair of networks

In pairs of species networks that shared dietary carotenoids, differences in network connectivity accounted for more of the metabolic distance between species’ networks (Fig. 7a; b_ST_ = 0.67, *t* = 75.24, *P* < 0.001, *n* = 4,839) than did differences in the diameters of the networks (Fig. 7b; b_ST_ = 0.28, *t* = 31.50, *P* < 0.001, *n* = 4,839). Pairs of networks with large differences in the average number of reactions per compound were more metabolically distinct than networks with large differences in their diameters.

### Structural properties of realized diversification of enzymatic reactions

**Fig. 8:**
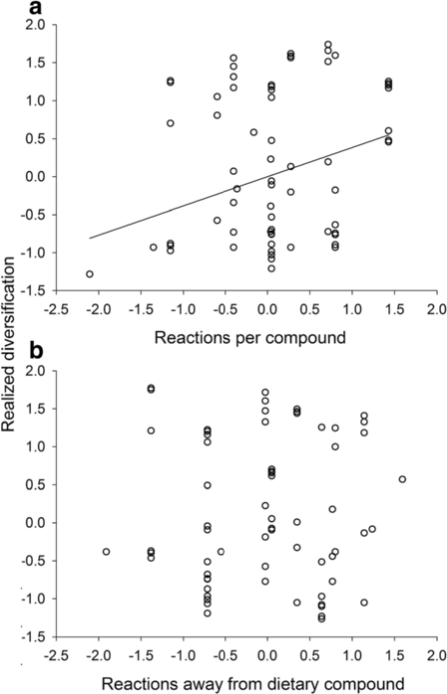
Realized diversification of the reactions associated with a compound (the fraction of species that do not have a reaction among all of the species that have the substrate compound for the reaction) was predicted by the connectivity of the substrate compound (reactions per compound), but not by the substrate compound’s distance from a dietary compound. Shown are partial regressions of the realized diversification of a reaction on (**a**), the enzymatic connectivity and (**b**), the distance from a dietary compound of the reaction’s substrate compound

The connectivity of a substrate compound contributed to the realized diversification across species of the reactions associated with the substrate compound (Fig. 8a; *b*_*ST*_ = 0.38, *t* = 3.10, *P* = 0.003, *n* = 81). The realized diversification of reactions in the network was not predicted by the distance of their substrate compounds from dietary compounds (Fig. 8b; *b*_*ST*_ = -0.05, *t* = -0.39, *P* = 0.70, *n* = 81).

## Discussion

To what extent is the exploration of a deterministic network and its associated phenotypic diversification the result of the network’s structural properties? The divergence between species’ networks could be driven by either the exploration of pathways from conserved compounds, the elongation of conserved pathways, or the addition of different modules. Our findings suggest that pathway diversification is the main mechanism of divergence among species’ metabolic networks; differences in the enzymatic connectivity among species’ networks contributed more to their metabolic divergence than did differences in the length of their diameters (Fig. 7). In the avian subset of the global carotenoid metabolic network, the connectivity of a compound strongly contributed to further network diversification: compound connectivity contributed the most to both the frequency of compound occurrence across species (Fig. 5a) and the realized diversification of the reactions associated with the compound among species’ networks (Fig. 8a). In contrast, pathway elongation did not play a major role in the diversification of avian carotenoid networks: the relative distance from a dietary compound was not related to a compound’s representation across species (Fig. 5b) or to the realized diversification of reactions associated with the compound among species’ networks (Fig. 8b). The presence of distinct structural modules and differences in the species representation of compounds within these modules contributed to the metabolic divergence across species: the most densely connected modules were the most prevalent across species’ networks. Metabolic divergence across species, however, was not due to the concurrent gain or loss of all of the compounds in a module (Fig. 6c and d). Thus, pathway diversification strongly contributes to metabolic divergence among species: modules characterized by greater connectivity provided more opportunities for the use of distinct pathways.

A central assumption of these tests and their interpretation, is that species are co-opting elements (genes or enzymes) that comprise the global avian carotenoid metabolic network and are selectively expressing a particular subset of these elements, rather than evolving them *de novo*. Several lines of evidence support this assumption. First, there was no correspondence between the historical relationships across study species and their utilization of carotenoid network space (i.e., use or disuse of particular reactions and compounds; [66, 79]). Instead the structure of networks, in particular the link between pathway elongation and pathway diversification, accounted for recurrent convergence of phylogenetically distant and ecologically distinct species in the utilization of network space and expression of carotenoid compounds (ibid.). Although such a pattern could be produced by the independent evolution of enzymes with identical functions, it is highly unlikely (e.g., [80]). In other taxa, horizontal gene transfer [58, 81–84] and symbiotic events [85] accounted for enzymatic convergence in carotenoid metabolism between unrelated species, but neither of these processes play a significant role in avian carotenoid biosynthesis. Gene duplications could similarly account for the evolution of convergent enzymes [24, 83, 86, 87], but the rate of gene duplications in birds [88] seems orders of magnitude lower that would be required to explain the documented rates of carotenoid enzyme convergence across bird species [66]. Instead, species-specific expression of compounds and reactions by the selective expression of different enzyme-encoding genes from the global carotenoid network, appears to be the dominant mode of avian carotenoid network evolution [88, 89], with *de novo* evolution of new carotenoid pathways (e.g., [90–92]) playing a secondary role (Additional file 1b). A potential mechanism that could drive pathway diversification of enzymatic reactions at these connected compounds is differences in the control of metabolic flux among species across different pathways [93]. Alternatively, different threshold concentrations of a substrate compound associated with several enzymatic reactions may be required to activate different enzymatic reactions [94, 95], such that the diversification of these pathways among species should be dependent on changes in the concentrations of these connected compounds.

We showed that the evolutionary representation of compounds and enzymatic reactions reflected their structural properties in the global carotenoid network (Fig. 5a). Why do compounds with the greatest connectivity tend to be overrepresented across species? The longer evolutionary persistence of the most connected elements is a common property of protein and gene deterministic networks across many taxa [e.g., 23, 24, 39, 40] and could reflect their role in maintaining the overall structural cohesiveness and function of the network. The removal or modification of highly connected elements could have greater pleiotropic effects that are more harmful to the function of the network than the removal of less connected compounds [96–98]. This property can result in stronger selection against the loss of these elements (e.g., [99]) or, alternatively, in lesser effectiveness of purifying selection for the deletion of centrally located elements in the network [100, 101]. Further, metabolic flux theory suggests that enzymes with the highest flux control coefficients should be located at the branching points of pathways in metabolic networks [102–105]. Such enzymes experience stronger stabilizing selection than those that contribute less to the flow of metabolites through metabolic pathways (e.g., [106]), accounting for the link between enzymatic connectivity and evolutionary persistence found in this study (Fig. 5a). These conclusions are corroborated by the models of network evolution and empirical studies of network growth that find that new elements in a network preferentially attach to evolutionarily stable elements that have greater connectivity rather than to sparsely connected, but more evolutionary labile downstream elements [24, 28, 34, 38].

It is possible that dietary compounds – the upstream-most elements of avian carotenoid networks – are not evolutionarily stable enough to contribute to incremental pathway elongation over evolutionary time. The evolutionary rates of the gain and loss of dietary carotenoids were orders of magnitude higher than the evolutionary lability of other compounds across avian metabolic networks [66], and our results show that dietary compounds were no more likely to be present in a network than metabolized downstream compounds (Fig. 5b). Theory predicts that rate-limiting enzymes should occur at upstream positions in pathways (e.g., [44]), however the evolutionary instability of dietary compounds can decrease the effectiveness of selection on these compounds. Instead, due to the high enzymatic connectivity of some compounds in carotenoid networks, pathways from different dietary starting points can ultimately produce the same end products (Fig. 2). Thus, network robustness to evolutionary labile dietary compounds – a central feature of avian carotenoid networks [66, 107] – may also contribute to the evolutionary stability of the connected compounds and explain why the diversification of species’ networks was centered on connected compounds instead of the continued lengthening of pathways from specific dietary compounds.

Variance in the species representations of compounds and enzymatic reactions within the same modules (Fig. 6c and d) implies that the modules partitioned by their structural properties do not correspond to actual biological processes (e.g. [108]), despite the fact that the structural modules used in this study were associated with different dietary compounds. Differences in the number of species with each compound in a module, however, could be the result of the connectivity of each of the compounds to other modules, which has been shown to explain the evolutionary rate of genes in protein interaction networks [109]. Furthermore, it is possible that species utilize all of the enzymatic reactions and produce all of the compounds in a module but selectively express only some of the compounds in their plumage [107, 110–112], and so the variation of the species representations of compounds in modules captures this selective compound deposition of the products of a module.

By identifying the topological structural properties in a deterministic network that underlie phenotypic differences we can begin to establish specific mechanisms for the microevolutionary sequences behind observed macroevolutionary patterns. For example, if highly connected network elements determine phenotypic differences, then phenotypic diversification in a lineage might not occur in sequential order (structural or temporal) because different pathways can be explored from the same initial conserved element, and so we would expect weak phylogenetic signal among phenotypes. If pathway elongation is the source of phenotypic differences, then the dependence between downstream and upstream elements imposes a clear sequential order to phenotypic diversification along the pathway, resulting in stronger historical associations across species’ networks. The incorporation or loss of entire modules of elements in a deterministic network may be ordered or unordered, depending on their relative positions, but either would result in recurrent bursts of diversification across lineages’ phenotypes [113–115]. Because we found no evidence of avian carotenoid network diversification due to pathway elongation, we would not expect a sequential order in patterns of realized diversification in carotenoid pathways during avian evolutionary history. Instead, our finding that differences among species’ networks were due to pathway diversification from highly connected compounds, suggests that related species should have similar carotenoid networks only when they utilize the same pathways from the same shared compound. The results of this study thus explain why phenotypic diversification in expressed carotenoids between related species was overwhelmingly due to unordered periodic bursts of biochemical diversification of several compounds at once in the same pathway module across species, with ecological divergence in the use of dietary carotenoids – the process closely associated with ecological speciation, pathway elongation, and species relatedness – playing a significantly weaker role [66, 107].

## Conclusions

The goal of this study was to explicitly consider how the structural interactions among elements of a trait affect its diversification. Our results show that the structure of the enzymatic reactions in the avian space of the global carotenoid network delineates opportunities for diversification of expressed carotenoids in birds. Within-species studies can establish the proximate mechanisms underlying the observed association of network topology, enzymatic connectivity and evolutionary diversification in carotenoid compounds. Explicit consideration of spatial and temporal organization of interactions between genes, proteins, enzymes and other elements of deterministic networks brings us closer to an understanding of the relationship between potential and realized phenotypic diversity.

## Abbreviation

MYA, million years ago

## Additional files

Additional file 1: (**a**) Appendix S1: Confirmed enzymatic reactions in the “avian space” of global carotenoid biosynthesis network in bacteria, plants, and animals. This appendix contains references supporting the presence of specific compounds and the enzymatic reactions that comprise the avian carotenoid biosynthesis global network. (**b**) Appendix S2: Characteristics of carotenoid metabolic networks for species used in the study. This appendix contains the structural measurements and references for compound identification and the method of identification for each of the species’ metabolic networks. (**c**) Appendix S3: Module assignments in the avian subset of the global carotenoid metabolic network. This appendix contains the module assignments for each of the compounds in the global avian carotenoid metabolic network. The number of the module corresponds to the partitioned regions in Fig. 2. (PDF 1501 kb) Additional file 2: Species’ binary metabolic networks. This appendix contains binary metabolic networks for each of the species included in the study. (XLSX 123 kb) Additional file 3: Majority rule consensus phylogeny of species included in the study. This appendix contains the Newick tree format of the majority rule consensus phylogeny visually presented in Fig. 4, 5, 6, 7 and 8. The tree is based on 1,000 randomly sampled trees from the Hackett All Species pseudo-posterior distribution downloaded from birdtree.org that is based on Jetz et al. 2012. (TXT 20 kb)
