# Supplementary material for: Structuring evolution: biochemical networks and metabolic diversification in birds
Source: BMC Evol Biol. 2016 Aug 25;16(1):168. doi: 10.1186/s12862-016-0731-z (PMC5000421; doi:10.1186/s12862-016-0731-z)
Supplement: Additional file 1: — (a) Appendix S1: Confirmed enzymatic reactions in the “avian space” of global carotenoid biosynthesis network in bacteria, plants, and animals. This appendix contains references supporting the presence of specific compounds and the enzymatic reactions that comprise the avian carotenoid biosynthesis global network. (b) Appendix S2: Characteristics of carotenoid metabolic networks for species used in the study. This appendix contains the structural measurements and references for compound identification and the method of identification for each of the species’ metabolic networks. (c) Appendix S3: Module assignments in the avian subset of the global carotenoid metabolic network. This appendix contains the module assignments for each of the compounds in the global avian carotenoid metabolic network. The number of the module corresponds to the partitioned regions in Fig. 2. (PDF 1501 kb) [file 12862_2016_731_MOESM1_ESM.pdf]

Appendix S1. Confirmed enzymatic reactions in the "avian space" of the global carotenoid biosynthesis network in bacteria, plants, and animals. Only nodes documented in birds are included.

			Bacteria					Algae					Plants					Animals				
CAROTENOID	Origin node	Reaction	NODE	PATH	ENZ	ISO	References	NODE	PATH	ENZ	ISO	References	NODE	PATH	ENZ	ISO	References	NODE	PATH	ENZ	ISO	References
**lutein**	1		?	.	.	.	70	Y	.	.	.	63, 70, 74	Y	.	.	.	63, 70, 74	Y	.	.	.	8, 9, 32, 33, 34, 37, 38, 39, 40, 41, 46, 86, 34, 91
	1	1-67	.	N	N	N	.	.	N	N	N	.	.	N	N	N	.		?	?	Y	46, 34, 93
	1	1-70	.	N	N	N	.	.	N	N	N	.	.	N	N	N	.	.	Y	Y	Y	8, 9, 33, 34, 42, 34, 107, 118
	1	1-52	.	N	N	N	.	.	N	N	N	.	.	?	N	Y	.	.	Y	N	Y	34, 91
	1	1-16	.	N	N	N	.	.	N	N	N	.	.	N	N	N	.	.	Y	Y	N	8, 10, 11, 33, 34, 39, 46, 34, 91, 93
	1	1-5	.	N	N	N	.	.	N	N	N	.	.	N	N	N	.	.	Y	Y	N	37, 41
	1	1-6	.	N	N	N	.	.	Y	Y	N	105	.	N	N	N	.	.	Y	Y	N	32, 38, 39, 40
	1	1-8	.	N	N	N	.	.	N	N	N	.	.	N	N	N	.	.	Y	Y	N	8, 10, 30, 51, 104, 123
	1	1-9	.	N	N	N	.	.	N	N	N	.	.	N	N	N	.	.	?	?	N	8, 30, 104
	1	1-13	.	N	N	N	.	.	N	N	N	.	.	N	N	N	.	.	Y	Y	N	9, 39, 42, 118
	1	1-51	.	N	N	N	.	.	N	N	N	.	.	N	N	N	.	.	Y	Y	n	9, 12, 39, 42, 118
	1	1-45	.	N	N	N	.	.	N	N	N	.	.	N	N	N	.	.	N	N	N	.
	1	1-46	.	Y	Y	N	67	.	N	N	N	.	.	Y	Y	N	67	.	.	.	N	.
**(3R, 3'R) zeaxanthin**	2		Y	.	.	.	2,14, 22, 62	Y	.	.	.	26, 62, 63, 70	Y	.	.	.	62, 44, 63, 74	Y	.	.	.	9,10, 11, 16, 29, 32, 38, 39, 44, 46, 54, 86
	2	2-69	.	N	N	N	.	.	N	N	N	.	.	?	?	N	70	.	Y	Y	N	46
	2	2-70	.	N	N	N	.	.	N	N	N	.	.	N	N	N	.	.	?	Y	Y	9, 33, 34, 42, 34
	2	2-68	.	N	N	N	.	.	N	N	N	.	.	N	N	N	.	.	Y	Y	N	46, 54, 86
	2	2-67	.	N	N	N	.	.	?	?	N	.	.	N	N	N	.	.	?	Y	Y	33, 44, 46, 54
	2	2-20	.	Y	Y	N	97	.	N	N	N	.	.	N	N	N	.	.	Y	Y	N	32, 35, 38, 39, 40, 71, 82
	2	2-16	.	N	N	N	.	.	N	N	N	.	.	N	N	N	.	.	Y	Y	N	9, 10, 32, 42, 46, 34
	2	2-21	.	N	N	N	.	.	Y	Y	N	26, 63	.	Y	Y	N	63	.	Y	Y	N	15
	2	2-31	.	N	N	N	.	.	N	N	N	.	.	N	N	N	.	.	Y	Y	N	9, 12, 42, 119, 120
	2	2-65	.	N	N	N	.	.	N	N	N	.	.	N	N	N	.	.	Y	Y	N	29, 46, 71
	2	2-32	.	Y	Y	N	2, 14	.	Y	Y	N	63	.	N	N	N	.	.	Y	Y	Y	9, 42, 70, 114
	2	2-4	.	N	N	N	.	.	N	N	N	.	.	N	N	N	.	.	Y	Y	N	54, 121
	2	2-18	?	?	?	N	.	.	?	?	N	.	.	N	N	N	.	.	?	?	N	8, 111, 119
**β-carotene**	3		Y	.	.	.	14, 22, 23, 59, 62, 75	Y	.	.	.	60, 61	Y	.	.	.	62, 63, 70	Y	.	.	.	3, 9, 16, 18, 28, 56, 57, 65, 64, 69, 71, 77, 78, 93
	3	3-4	.	Y	Y	N	2, 14, 22, 23, 59, 62, 75	.	Y	Y	N	20, 61, 63	.	Y	Y	N	63, 96		Y	Y	N	57, 65, 69, 93
	3	3-35	.	Y	Y	N	2, 14, 22, 23, 59, 62, 75	.	Y	Y	N	20, 61, 63	.	N	N	N	.	.	Y	Y	N	1, 12, 9, 57, 65, 93
	3	3-41		Y	Y	N	81		Y	Y	N	70, 98	.	Y	Y	N	96		Y	Y	N	5, 16, 18, 28, 64, 77, 78, 83
**β-cryptoxanthin**	4		Y	.	.	.	2,14, 22,23, 59, 62, 75	Y	.	.	.	22, 61, 62, 63, 70	Y	.	.	.	62, 70	Y	.	.	.	9, 10, 65, 69, 56, 57, 71
	4	4-2		Y	Y	N	2,14, 22, 23, 59, 62	.	Y	Y	N	22, 62	.	Y	Y	N	62, 63	.	Y	Y	N	69
	4	4-36	.	Y	Y	N	2,14, 22, 23, 59, 62	.	Y	Y	N	22, 62	.	N	N	N	.	.	Y	Y	N	57, 93
	4	4-30	.	Y	Y	N	97	.	?	?	N	.	.	N	N	N	.	.	Y	Y	N	32, 39, 40
**anhydrolutein**	5		N	.	.	.	.	N	.	.	.	.	N	.	.	.	.	Y	.	.	.	32, 37, 41
**7,8- dihydrolutein**	6		N	.	.	.	.	Y	.	.	.	105	N	.	.	.	.	Y	.	.	.	32, 39, 40, 82
	6	6-7	.	N	N	N	.		?	N	Y	105	.	N	N	Y	.	.	Y	N	Y	32, 39, 40, 82
	6	6-109	.	N	N	N	.	.	N	N	N	.	.	N	N	N	.	.	?	?	N	201
**9-Z-7,8-dihydrolutein**	7		N	.	.	.	.	?	.	.	.	.	N	.	.	.	.	Y	.	.	.	32, 39, 40, 82
	7	7-6	.	N	N	N	.	.	?	N	Y	105	.	N	N	Y	.	.	Y	N	Y	32, 39, 40, 82
**canary xanthophyll A**	8		N	.	.	.	.	N	.	.	.	.	N	.	.	.	.	Y	.	.	.	8, 9, 10, 11, 17, 32, 33, 46, 71, 91, 34, 104
	8	8-10	.	N	N	N	.	.	N	N	N	.	.	N	N	N	.	.	Y	Y	N	9, 11, 32, 33, 39, 34
	8	8-16	.	N	N	N	.	.	N	N	N	.	.	N	N	N	.	.	Y	Y	N	34, 91
	8	8-9	.	N	N	N	.	.	N	N	N	.	.	N	N	N	.	.	Y	Y	N	9, 10, 11, 32, 33, 39, 34, 104, 123
	8	8-110	.	N	N	N	.	.	N	N	N	.	.	N	N	N	.	.	?	?	N	202
**canary xanthophyll B**	9		N	.	.	.	.	N	.	.	.	.	N	.	.	.	74	Y	.	.	.	8, 9, 11, 10, 17, 32, 33, 46, 71, 104
	9	9-17	.	N	N	N	.	.	N	N	N	.	.	N	N	N	.	.	?	?	N	36
	9	9-8	.	N	N	N	.	.	N	N	N	.	.	N	N	N	.	.	Y	Y	N	9, 11
**(3S, 6S,3'S, 6'S) tunaxanthin A**	10		N	.	.	.	.	N	.	.	.	.	?	.	.	.	.	Y	.	.	.	8, 11, 32, 33, 34, 39, 71, 34
	10	10-11	.	N	N	N	.	.	N	N	N	.	.	?	.	.	.		Y	N	Y	8, 11, 39, 71, 108, 111
**(3R, 6R,3'R, 6'R) tunaxanthin F**	11		N	.	.	.	.	N	.	.	.	.	Y	.	.	.	47, 48	Y	.	.	.	8, 91
	11	11-66	.	N	N	N	.	.	N	N	N	.	.	N	N	N	.	.	?	?	N	8
	11	11-65	.	N	N	N	.	.	N	N	N	.	.	N	N	N	.	.	?	?	N	8
**α-doradexanthin**	12		N	.	.	.	.	Y	.	.	.	70	N	.	.	.	.	Y	.	.	.	39, 42, 45, 70
	12	12-14	.	N	N	N	.	.	N	N	N	.	.	N	N	N	.	.	N	N	N	130
	12	12-15	.	N	N	N	.	.	N	N	N	.	.	N	N	N	.	.	Y	Y	N	45, 111
	12	12-32	.	N	N	N	.	.	N	N	N	.	.	N	N	N	.	.	Y	Y	N	71, 117
	12	12-100	.	N	N	N	.	.	N	N	N	.	.	N	N	N	.	.	?	?	N	200
**(3S,4R,3'R,6'R) 4-hydroxylutein**	13		N	.	.	.	.	N	.	.	.	.	N	.	.	.	.	Y	.	.	.	9, 39, 42
	13	13-51	.	N	N	N	.	.	N	N	N	.	.	N	N	N	.	.	Y	N	N	42, 111
	13	13-14	.	N	N	N	.	.	N	N	N	.	.	N	N	N	.	.	Y	?	N	9, 12, 39, 42, 111, 118, 130
**fritschiellaxanthin**	14		N	.	.	.	.	Y	.	.	.	70	N	.	.	.	.	Y	.	.	.	9, 39, 42, 70
	14	14-12	.	N	N	N	.	.	?	N	?	.	.	?	N	Y	.	.	N	N	N	.
	14	14-15	.	N	N	N	.	.	N	N	N	.	.	N	N	N	.	.	Y	?	N	9, 111
**papilioerythrinone**	15		N	.	.	.	.	Y	.	.	.	.	N	.	.	.	.	Y	.	.	.	45, 71
	15	15-108	.	N	N	N	.	.	N	N	N	.	.	N	N	N	.	.	?	?	N	201
**3'-dehydrolutein**	16		N	.	.	.	.	?	.	.	.	.	?	.	.	.	.	Y	.	.	.	8, 9, 10, 11, 33, 34, 35, 46, 71, 34, 91, 93
	16	16-1	.	N	N	N	.	.	?	.	.	.	.	N	N	N	.	.	Y	Y	N	9, 11, 34, 91
	16	16-8	.	N	N	N	.	.	N	N	N	.	.	N	N	N	74		N	Y	N	10, 11, 34, 92

	16	16-9	.	N	N	N	.	.	N	N	N	.	.	N	N	N	.	.	Y	Y	N	10, 11, 34
	16	16-70	.	N	N	N	.	.	N	N	N	.	.	N	N	N	.	.	Y	Y	N	33, 34, 91
	16	16-109	.	N	N	N	.	.	N	N	N	.	.	N	N	N	.	.	?	?	N	201
**piprixanthin**	17		N	.	.	.	.	N	.	.	.	.	N	.	.	.	.	Y	.	.	.	36
	17	17-71		N	N	N	.	.	N	N	N	.	.	N	N	N		.	?	?	N	36
	17	17-18		Y	N	N	.	.	?	N	N	.	.	N	N	N	.	.	Y	?	N	36
**rhodoxanthin**	18		Y	.	.	.	70	Y	.	.	.	.	Y	.	.	.	70	Y	.	.	.	36
**7,8,7',8'-tetrahydrozeaxanthin**	19		N	.	.	.	.	N	.	.	.	.	N	.	.	.	.	Y	.	.	.	32, 35, 40, 82
**7,8-dihydrozeaxanthin**	20		Y	Y	.	.	59, 97	N	.	.	.	.	N	.	.	.	.	Y	.	.	.	32, 38, 39, 40, 71
	20	20-19	.	N	N	N	.	.	N	N	N	.	.	N	N	N	.	.	Y	Y	N	32, 35, 40, 82
**antheraxanthin**	21		N	.	.	.	.	Y	.	.	.	26, 63, 70	Y	.	.	.	62, 63, 70, 74	Y	.	.	.	9, 71
	21	21-22	.	N	N	N	.	.	Y	Y	N	63	.	Y	Y	N	63, 74	.	N	N	N	.
	21	21-2	.	N	N	N	.	.	Y	Y	N	63	.	Y	Y	N	63, 74, 75	.	N	N	N	.
	21	21-76	.	N	N	N	.	.	?	?	N	.	.	Y	Y	N	71, 115	.	N	N	N	.
**violaxanthin**	22		N	N	.	N	.	Y	.	.	.	26, 63, 70	Y	.	.	.	62, 63, 70, 74	Y	.	.	.	71
	22	22-23	.	N	N	N	.	.	Y	Y	N	26, 63, 70	.	Y	Y	N	62, 63, 70, 74		N	N	.	.
	22	22-21	.	N	N	N	.	.	Y	Y	N	26, 63, 70	.	Y	Y	N	62, 63, 70, 74		N	N	.	.
**neoxanthin**	23		N	.	.	.	.	Y	.	.	.	26, 63	Y	.	.	.	62, 63, 70, 74, 105	Y	.	.	.	49, 71
	23	23-24	.	N	N	N	.	.	N	N	N	.	.	?	?	N	.	.	Y	Y	N	49
**neochrome**	24		N	.	.	.	.	Y	.	.	.	105, 112	Y	.	.	.	70	Y	.	.	.	49
**idoxanthin**	25		N	.	.	.	.	?	.	.	.	111	N	.	.	.	.	Y	.	.	.	9,11, 13, 15, 16, 24, 28, 71
	25	25-34	.	N	N	N	.	.	N	N	N	.	.	N	N	N	.	.	Y	?	N	120, 126, 127
	25	25-32	.	N	N	N	.	.	N	N	N	.	.	.	N	N	.	.	Y	Y	N	11, 13, 15, 16, 35
	25	25-74	.	N	N	N	.	.	N	N	N	.	.	N	N	N	.	.	Y	Y	N	11, 71
**fucoxanthin**	26		N	.	.	.	.	Y	.	.	.	70	N	.	.	.	.	Y	.	.	.	5, 51, 88, 71
	26	26-27	.	N	.	N	.	.	N	N	.	.	.	N	N	N	.		Y	Y	N	5, 51, 88, 122
**fucoxanthinol**	27		N	.	.	.	.	Y	.	.	.	70, 124	N	N	N	N	.	Y	.	.	.	5, 51, 88, 71
	27	27-28	.	N	N	N	.	.	N	N	N	.	.	N	N	N	.	.	Y	Y	N	5, 51
	27	27-29	.	N	N	N	.	.	N			.	.	N	N	N	.	.	?	?	N	122
**amarouciaxanthin**	28		N	.	.	.	.	N	.	.	.	.	N	.	.	.	.	Y	.	.	.	51
	28	28-29	.	N	N	N	.	.	N	N	N	.	.	N	N	N	.		Y	Y	N	5, 51, 88
**paracentrone**	29		N	.	.	.	.	?	.	.	.	112	N	.	.	.	.	Y	.	.	.	5, 88
**7,8 dihydro β-cryptoxanthin**	30		Y	.	.	.	97	?	.	.	.	97	?	.	.	.	97	Y	.			32, 39, 40
**4-hydroxyzeaxanthin**	31		N	.	.	.	.	N	.	.	.	.	N	N	N	N	.	Y	.	.	.	8, 9, 16, 11, 71
	31	31-32	.	N	N	N	.	.	N	N	N	.	.	N	N	N	.	.	Y	?	N	9, 12, 42
	31	31-2	.	N	N	N	.	.	N	N	N	.	.	N	N	N	.	.	Y	Y	N	8, 9, 11
	31	31-74	.	N	N	N	.	.	N	N	N	.	.	N	N	N	.	.	Y	Y	N	42, 120
**adonixanthin**	32		Y	.	.	.	2, 14, 22, 59	Y	.	.	.	20, 61, 63	Y	.	.	.	80, 96, 125	Y	.	.	.	9, 11, 15, 16, 28, 29, 32, 35, 42
	32	32-34	.	Y	Y	N	2, 14, 22, 59	.	Y	Y	N	20, 61, 63	.	N	N	N	.	.	Y	Y	N	9, 12, 35, 104, 118
	32	32-2	.	N	N	N	.	.	N	N	N	.	.	N	N	N	.	.	Y	Y	N	9, 16, 35, 39
	32	32-25	.	N	N	N	.	.	N	N	N	.	.	N	N	N	.	.	Y	Y	N	9, 42, 70
	32	32-31	.	N	N	N	.	.	N	N	N	.	.	N	N	N	.	.	Y	Y	N	8, 9, 11, 16
	32	32-102	.	N	N	N	.	.	N	N	N	.	.	N	N	N	.	.	?	?	N	200
**13 cis-(3R, 3'R) astaxanthin**	33		Y	.	.	.	73	Y	.	.	.	84	?	.	.	.	.	Y	.	.	.	15, 71, 85
	33	33-34	.	Y	N	Y	73	.	Y	N	Y	84	.	?	N	Y	.	.	Y	N	Y	15, 27, 71, 85
**(3'R, 3R) astaxanthin**	34		Y	.	.	.	2, 14, 22, 59, 102	Y	.	.	.	20, 63, 70	Y	.	.	.	74, 96	Y	.	.	.	8, 11, 13, 15, 16, 27, 29, 35, 45, 57, 71
	34	34-32	.	N	N	N	.	.	N	N	N	.	.	N	N	N	.	.	Y	Y	N	8, 9, 11, 15, 29
	34	34-25	.	N	N	N	.	N	N	N	N	.	.	N	N	N	.	.	Y	Y	N	8, 11, 13, 15, 27
	34	34-75	.	Y	N	Y	73	.	Y	N	Y	84	.	?	N	Y	.	.	Y	N	Y	15, 71, 85
	34	34-38	.	N	N	N	.	.	N	N	N	.	.	N	N	N	.	.	Y	Y	N	121
	34	34-33	.	Y	Y	Y	73	.	Y	N	Y	84	.	?	N	Y	.	.	Y	N	Y	15, 27, 71, 85
	34	34-103	.	N	N	N	.	.	N	N	N	.	.	N	N	N	.	.	?	?	N	200
**echinenone**	35		Y	.	.	.	2, 14, 22, 23, 25, 59, 62, 63, 75, 100	Y	.	.	.	61, 63, 70	Y	.	.	.	80, 96	Y	.	.	.	15, 16, 28, 64, 71, 77, 78, 83, 93
	35	35-3	.	N	N	N	.	.	N	N	N	.	.	N	N	N	.	.	Y	Y	N	4, 15, 16
	35	35-39	.	Y	Y	N	81, 100	.	Y	Y	N	.	.	N	N	N	.	.	Y	Y	N	6, 28, 64, 77, 78
	35	35-37	.	Y	Y	N	2, 14, 22, 59, 62, 102	.	Y	Y	N	62, 63	.	N	N	N	.	.	Y	Y	N	2, 9, 57, 64, 93
	35	35-36	.	Y	Y	N	2, 14, 22, 23, 59, 62, 63, 102	.	Y	Y	N	62, 63	.	Y	Y	N	80, 96, 125	.	Y	Y	N	57, 71, 101, 93
	35	35-41	.	N	N	N	.	.	N	N	N	.	.	N	N	N	.	.	Y	Y	N	16
**3'-hydroxyechinenone**	36		Y	.	.	.	2, 14, 22, 59, 63, 102	Y	.	.	.	20, 63, 70	Y	.	.	.	80, 96	Y	.	.	.	15, 17, 57, 71, 93
	36	36-32	.	Y	Y	N	2, 14, 22, 59, 102	.	Y	Y	N	20, 63	.	Y	Y	.	80, 96, 125	.	Y	?	N	57, 71, 93
	36	36-38	.	Y	Y	N	2,14, 59, 102	.	Y	Y	N	20, 63	.	N	N	N	.	.	Y	Y	N	57, 71, 93
	36	36-4	.	N	N	N	.	.	N	N	N	.	.	N	N	N	.	.	Y	Y	N	9, 15
**canthaxanthin**	37		Y	.	.	.	2,14,22, 25, 59, 63, 81	Y	.	.	.	61, 63, 70	Y	.	.	.	.	Y	.	.	.	3, 15, 16, 28, 57, 64, 77, 78, 93
	37	37-35	.	N	N	N	.	.	N	N	N	.	.	N	N	N	.	.	Y	Y	N	4, 8, 15, 121
	37	37-38	.	Y	Y	N	2, 14, 22, 59, 63, 102	.	Y	Y	N	62, 63	.	Y	Y	N	80, 96	.	Y	Y	N	9, 17, 57, 93, 101, 121
	37	37-40	.	N	N	N	.	.	N	N	N	.	.	N	N	N	.	.	Y	Y	N	8, 16
	37	37-39	.	N	N	N	.	.	N	N	N	.	.	N	N	N	.	.	Y	Y	N	15, 16, 31, 82
**adonirubin**	38		Y	.	.	.	2,14, 22, 59, 102	Y	.	.	.	20, 63, 70	Y	.	.	.	96	Y	.	.	.	1, 3, 6, 7, 17, 57, 71, 101
	38	38-34	.	Y	Y	N	2, 14, 22, 59, 102	.	Y	Y	N	63	.	Y	Y	N	74, 80, 96, 125	.	Y	Y	N	9, 17, 57, 93, 101, 71, 77, 103
	38	38-36	.	N	N	N	.	.	N	N	N	.	.	N	N	N	.	.	Y	Y	N	15
	38	38-37	.	Y	Y	N	89	.	Y	Y	N	62, 63	.	Y	Y	N	80, 96	.	Y	Y	N	89, 75
	38	38-107	.	N	N	N	.	.	N	N	N	.	.	N	N	N	.	.	?	?	N	200
**4-hydroxy-echinenone**	39		Y	.	.	.	81, 100	Y	.	.	.	70	N	.	.	.	.	Y	.	.	.	6, 16, 30, 31, 71, 77, 78, 82, 83, 99
	39	39-40	.	N	N	N	.	.	N	N	N	.	.	N	N	N	.	.	Y	Y	N	15, 16, 31
	39	39-35	.	N	N	N	.	.	N	N	N	.	.	N	N	N	.	.	Y	Y	N	16

	39	39-37	.	Y	?	N	81, 100	.	?	?	N	.	.	N	N	N	.	.	Y	Y	N	6, 28, 35, 64, 77, 78, 83
**isozeaxanthin**	40		Y	.	.	.	81	Y	.	.	.	70, 98	Y	.	.	.	80	Y	.	.	.	8, 16, 28, 31, 53, 71, 80, 81
	40	40-41	.	N	N	N	.	.	N	N	.	.	.	N	N	N	.	.	Y	Y	N	8, 15, 16
	40	40-60	.	N	N	N	.	.	N	N	.	.	.	Y	Y	N	80	.	N	N	N	.
	40	40-37	.	Y	Y	N	81	.	Y	Y	N	70, 98	.	N	N	N	.	.	Y	Y	N	28, 64
	40	40-39	.	Y	Y	N	81	.	?	?	N	70	.	N	N	N	.	.	N	N	N	.
**β-isocryptoxanthin**	41		Y	.	.	.	81	Y	.	.	.	79, 98	Y	.	.	.	70	Y	.	.	.	5, 9, 18, 16, 31, 71, 77, 78, 83
	41	41-3	.	N	N	N	.	.	N	.	N	.	.	N	N	N	.		N	Y	N	16
	41	41-40	.	Y	Y	N	81		Y	Y	N	70, 98	.	Y	Y	N	96	.	Y	Y	N	28, 71
	41	41-35	.	?	Y	N	81	.	?	?	N	70, 98	.	N	N	N	.	.	Y	Y	N	18, 28, 64, 77, 78, 83
**α-carotene**	42		Y	N	.	.	62, 63, 70, 76	Y	.	.	.	62, 63, 70, 74	Y	.	.	.	58, 59, 63, 74, 76	Y	.	.	.	52, 53, 71
	42	42-46	.	N	N	N	.	.	N	N	N	.	.	Y	Y	N	131	.	?	?	N	.
	42	42-45	.	?	Y	N	59	.	Y	Y	N	63	.	Y	Y	N	63	.	N	N	.	.
	42	42-43	.	N	N	N	.	.	N	N	N	.	.	?	?	N	96, 125	.	Y	?	.	72
**α-isocryptoxanthin**	43		?	.	.	.	.	?	.	.	.	.	?	.	.	.	.	Y	.	.	.	52, 71, 72
	43	43-44	.	N	N	N	.	.	N	N	N	.	.	N	N	N	.	.	Y	Y	N	52, 72
**phoenicopterone**	44		N	.	.	.	.	Y	.	.	.	70, 113	N	.	.	.	.	Y	.	.	.	53, 71, 72
**zeinoxanthin**	45		N	.	.	.	.	Y	.	.	.	58, 59, 63	Y	.	.	.	58, 59, 63	N	.	.	.	.
	45	45-1	.	N	N	N	.	.	Y	Y	N	63	.	Y	Y	N	63	.	N	N	N	.
	45	45-42	.	N	N	N	.	.	N	N	N	.	.	N	N	N	.	.	N	N	N	.
**α-cryptoxanthin**	46		N	.	.	.	.	Y	.	.	.	63, 70	Y	.	.	.	63, 70	Y	.	.	.	46, 71, 106
	46	46-1	.	Y	Y	N	67	.	N	N	N	.	.	Y	Y	N	67	.	.	.	N	.
	46	46-42	.	N	N	N	.	.	N	N	N	.	.	Y	Y	N	131	.	?	?	N	8
**rubixanthin**	47		Y	.	.	.	70, 94	Y	.	.	.	70, 95	Y	.	.	.	70	Y	.	.	.	55, 57, 93, 71
	47	47-48	.	Y	Y	N	.	.	?	Y	N	.	.	?	?	N	.	.	Y	Y	N	57
	47	47-49	.	?	?	Y	.	.	?	Y	Y	.	.	Y	N	Y	55	.	N	N	N	55, 104, 128
	47	47-2	.	Y	?	N	129	.	.	.	.	.	.	.	.	.	.	.	.	.	.	.
	47	47-4	.	Y	?	N	129	.	.	.	.	.	.	.	.	.	.	.	.	.	.	.
**4-oxo-rubixanthin**	48		?	.	.	.	.	?	.	.	.	.	?	.	.	.	.	Y	.	.	.	57, 93
	48	48-50	.	?	?	?	.	.	?	?	?	.	.	N	N	N	.	.	N	N	N	.
**gazaniaxanthin**	49		?	.	.	.	.	?	.	.	.	.	Y	.	.	.	70	Y	.	.	.	71, 104
	49	49-50	.	?	?	N	.	.	?	?	N	.	.	?	?	.	.	.	Y	Y	N	93, 104
	49	49-47	.	?	?	Y	.	.	?	?	Y	.		Y	Y	Y	55	.	N	N	N	55, 104, 128
**4-oxo-gazaniaxanthin**	50		?	.	.	.	.	?	.	.	.	.	?	.	.	.	.	Y	.	.	.	57, 93, 104
	50	50-48	.	?	?	?	.		?	?	?	.	.	N	N	N	.	.	?	?	Y	93
**(3S,4R,3'S,6'R) 4-hydroxylutein**	51		N	.	.	.	.	N	.	.	.	.	N	.	.	.	.	Y	.	.	.	9, 39, 42
	51	51-13	.	N	N	N	.	.	N	N	N	.	.	N	N	N	.	.	Y	N	N	42, 70, 111
	51	51-12	.	N	N	N	.	.	N	N	N	.	.	N	N	N	.	.	Y	Y	N	9, 12, 39, 42, 118
**cis lutein**	52		N	.	.	.	.	?	.	.	.	.	?	.	.	.	.	Y	.	.	.	34, 91
	52	52-1	.	N	N	N	.	.	?	N	N	.	.	?	N	Y	.	.	Y	N	Y	34, 91, 93
**3,4, 3',4'-tetradehydroisozeanthin**	60		N	.	.	.	.	N	.	.	.	.	Y	.	.	.	80	N	.	.	.	.
	60	60-37	.	N	N	N	.	.	N	N	N	.	.	Y	N	N	80	.	N	N	N	.
**γ-carotene**	61		Y	.	.	.	62, 73, 94	Y	.	.	.	63	Y	.	.	.	58, 62, 63, 70	Y	.	.	.	71, 72
	61	61-3	.	Y	Y	N	62, 73	.	Y	Y		63		Y	Y	N	62, 63, 74		N	N	N	.
	61	61-47		Y	Y	N	73, 94		?	?	N	.	.	?	Y	N	58, 70		?	?	N	.
	61	61-42	.	N	N	N	62	.	Y	Y	.	63	.	Y	Y	N	62, 63, 70	.	N	N	N	.
**δ-carotene**	64		Y	.	.	.	62	Y	.	.	.	63, 70	Y	.	.	.	63, 74	Y	.	.	.	71
	64	64-65	.	N	N	N	63, 76	.	Y	Y	N	62, 63	.	Y	Y	N	62, 63, 87		N	N	N	.
	64	64-42	.	Y	Y	N	62	.	Y	Y	N	62, 63	.	Y	Y	N	62, 63, 87		N	N	N	.
**(6S, 6'S) ε,ε- carotene**	65		N	.	.	.	.	Y	.	.	.	63, 70	Y	.	.	.	48, 63, 74, 87	Y	.	.	.	8, 39, 46, 71
	65	65-66	.	N	N	N	.	.	?	?	N	.	.	Y	Y	N	47	.	?	?	N	.
**ε-carotene 3 diol**	66		N	.	.	.	.	?	.	.	.	.	Y	.	.	.	47	Y	.	.	.	.
	66	66-65	.	N	N	N	.	.	N	.	N	.	.	N	N	N	.	.	?	?	N	8
	66	66-11	.	N	N	N	.	.	N	N	N	.	.	Y	Y	N	47	.	?	?	N	.
**(3R, 3'S) meso-zeaxanthin**	67		N	.	.	.	.	N	.	.	.	.	N	.	.	.	44	Y	.	.	.	16, 33, 44, 46, 54, 86, 34, 93
	67	67-2	.	N	N	N	.	.	N	N	N	.	.	N	N	N	.	.	?	?	Y	33, 44, 54
	67	67-1	N	N	N	N	.	.	N	N	N	.	.	N	N	N	.	.	Y	Y	Y	34, 93
**galloxanthin**	68		N	.	.	.	.	N	.	.	.	.	N	.	.	.	.	Y	.	.	.	29, 46, 54, 71
**β-apo-2'-carotenol**	69		?	Y	.	.	111	?	.	.	.	111	N	.	.	.	111	Y	.	.	.	46
**3'-epilutein**	70		N	.	.	.	.	?	.	.	.	112	?	.	.	.	.	Y	.	.	.	9, 11, 33, 34, 39, 71, 34
	70	70-16	.	N	N	N	.	.	N	N	N	.	.	N	N	N	.	.	?	?	N	33, 46, 34
	70	70-8	.	N	N	N	.	.	N	N	N	.	.	N	N	N	.	.	Y	Y	N	33
	70	70-1	.	N	N	.	.	.	?	?	N	.	.	N	N	N	.	.	?	?	Y	33, 34, 71, 34
	70	70-2	.	N	N	N	.	.	N	N	N	.	.	N	N	N	.	.	Y	Y	Y	33, 34, 34, 91
	70	70-12	.	N	N	N	.	.	N	N	N	.	.	N	N	N	.	.	?	?	N	93
	70	70-51	.	N	N	N	.	.	N	N	N	.	.	N	N	N	.	.	Y	Y	N	9, 12, 39, 42, 118
**resonance stabilized form**	71		?	.	.	.	.	?	.	.	.	.	N	.	.	.	.	Y	.	.	.	36
	71	71-18	.	?	N	N	.	.	?	N	N	.	.	N	N	N	.	.	?	?	N	36
**β-carotene -3,4, 3', 4'- tetrol**	74		N	.	.	.	.	Y	.	.	.	70	N	.	.	.	.	Y	.	.	.	11, 42, 71
	74	74-31	.	N	N	N	.	.	N	N	N	.	.	N	N	N	.	.	Y	Y	N	11
	74	74-25	.	N	N	N	.	.	N	N	N	.	.	N	N	N	.	.	Y	Y	N	11, 42, 120
**9-cis - (3S, 3'S) astaxanthin**	75		?	.	.	.	.	Y	.	.	.	84	?	.	.	.	.	Y	.	.	.	15, 71, 85
	75	75-34	.	Y	N	Y	73	.	Y	N	Y	84	.	?	N	Y	.	.	Y	N	Y	15, 27, 71, 85

**eschscholtzxanthin**	76		N	.	.	.	.	N	.	.	.	.	Y	.			70	Y	.	.	.	.
	76	76-77	.	N	N	N	.	.	N	N	N	.	.	Y	Y	N	111, 116	.	N	N	N	.
**eschscholtzxanthone**	77		N	.	.	.	.	N	.	.	.	.	Y	.	.	.	116	N	.	.	.	.
	77	77-18	.	N	N	N	.	.	N	N	N	.	.	Y	Y	N	116	.	N	N	N	.
**xipholenin (Note 1)**	100		N	.	.	.	.	N	.	.	.	.	N	.	.	.	.	Y	.	.	.	200, 203
	100	100-101	.	N	N	N	.	.	N	N	N	.	.	N	N	N	.	.	?	?	N	200, 203
**2,3-didehydro-xipholenin (Note 2)**	101		N	.	.	.	.	N	.	.	.	.	N	.	.	.	.	Y	.	.	.	200, 203
**rupicolin (Note 3)**	102		N	.	.	.	.	N	.	.	.	.	N	.	.	.	.	Y	.	.	.	200
**3'-hydroxy-3-methoxy-canthaxanthin**	103		N	.	.	.	.	N	.	.	.	.	N	.	.	.	.	?	.	.	.	200
	103	103-104	.	N	N	N	.	.	N	N	N	.	.	N	N	N	.	.	?	?	N	200
**pompadourin (Note 4)**	104		N	.	.	.	.	N	.	.	.	.	N	.	.	.	.	Y	.	.	.	200, 203
	104	104-105	.	N	N	N	.	.	N	N	N	.	.	N	N	N	.	.	?	?	N	200, 203
**2,3-Didehydro-pompadourin (Note 5)**	105		N	.	.	.	.	N	.	.	.	.	N	.	.	.	.	Y	.	.	.	200, 203
	105	105-106	.	N	N	N	.	.	N	N	N	.	.	N	N	N	.	.	?	?	N	200, 203
**cotingin (Note 6)**	106		N	.	.	.	.	N	.	.	.	.	N	.	.	.	.	Y	.	.	.	200, 203
**brittonxanthin (Note 7)**	107		N	.	.	.	.	N	.	.	.	.	N	.	.	.	.	Y	.	.	.	200, 203
**cymbirhynchin (Note 8)**	108		N	.	.	.	.	N	.	.	.	.	N	.	.	.	.	Y	.	.	.	201
**eurylaimin (Note 9)**	109		N	.	.	.	.	N	.	.	.	.	N	.	.	.	.	Y	.	.	.	201
**4-hydroxy-canary xanthophyll A**	110		N	.	.	.	.	N	.	.	.	.	N	.	.	.	.	Y	.	.	.	202

Y: confirmed present, N: confirmed absent, ?: expected, but no experimental evidence

NODE -- carotenoid compound (number refers to network)

PATH -- reaction from origin node to derived node

ENZ -- presence of enzyme

ISO -- evidence of isomerization

Notes:

1: 3-Methoxy-3'-hydroxy-β,ε-carotene-4-one

2: 3'-hydroxy-3-methoxy-2,3-didehydro-β,β-carotene-4-one

3: 3'-hydroxy-3-methoxy-β,β-carotene-4-one

4: 3,3'-Dimethoxy-β,β-carotene-4,4'-dione or 3,3'-dimethoxy-canthaxanthin

5: 3,3'-Dimethoxy-2,3-didehydro-β,β-carotene-4,4'dione

6: 3,3'-Dimethoxy-2,3,2',3'-tetradehydro-β,β-carotene-4,4'dione

7: 3-methoxy-β,β-carotene-4,4'dione or 3-methoxy-canthaxanthin

8: 2,3-didehydro-papilioerythrinone

9: 7,8-dihydro-3'-dehydro-lutein

Literature Sources for Appendix S1:

1. Davies, B. H., W. J. Hsu, and C. O. Chichester. 1970. The mechanism of the conversion of beta-carotene into canthaxanthin by the brine shrimp, Artemia salina L. (Crustacea: Branchiopoda). Comp. Biochem Physiol. 33: 601-615.

2. Fraser, P. D., S. Hiroshi, and M. Norihiko. 1998. Enzymic confirmation of reactions involved in routes to astaxanthin formation, elucidated using a direct substrate *in vitro* assay. Eur. J. Biochem. 252: 229-236.

3. Fox, D. L., A. A. Wolfson, and J. W. McBeth. 1969. Metabolism of □-carotene in the american flamingo, Phoenicopterus rubber. Comp. Biochem. Physiol. 29:1223-1229.

4. Guillou, A., G. Choubert, T. Storebakken, J. De La Noüe, and S. Kaushik. 1989. Bioconversion pathway of astaxanthin into retinol$_2$ in mature rainbow trout (Salmo gairdneri Rich.). Comp. Biochem. Physiol. 94: 484-485.

5. Hallenstvet, M., E. Pyberg, and S. Liaaen-Jensen. 1978. Animal carotenoids - XIV Carotenoids of Psammechinus miliaris (sea-urchin). Comp. Biochem. Physiol. 60: 173-175.

6. Hata, M., and Hata, M. 1969. Carotenoid metabolism in Artemia salina L. Comp. Biochem. Physiol. 29: 985-994.

7. Herring, P. J. 1968. The carotenoid pigments of Daphnia magna Straus. II. Aspects of pigmentary metabolism. Comp. Biochem. Physiol. 24: 205-221.

8. Katsuyama, M., and Matsuno,T. 1988. Carotenoid and vitamin A, and metabolism of carotenoids, □-carotene, canthaxanthin, astaxanthin, zeaxanthin, lutein and tunaxanthin in tilapia *Tilapia nilotica*. Comp. Biochem. Physiol. B 90: 131-139.

9. Matsuno,T. 1991. Xanthophylls as precursors of retinoids. Pure Appl. Chem. 63: 81-88.

10. Matsuno, T., T. Hirono, Y. Ikuno, T. Maoka, M. Shimizu, and T. Komori. 1986. Isolation of three new carotenoids and proposed metabolic pathways of carotenoids in hen's egg yolk. Comp. Biochem. Physiol. 84: 477-481.

11. Matsuno, T., M. Katsuyama, T. Maoka, T. Hirono, and T. Komori. 1985. Reductive metabolic pathways of carotenoids in fish (3S, 3'S)-astaxanthin to tunaxanthin A, B and C. Comp. Biochem. Physiol. 80:779-789.

12. Matsuno,T., H. Matsutaka, and S. Nagata. 1981. Metabolism of lutein and zeaxanthin to ketocarotenoids in goldfish, Carassius auratus. Bull. Jap. Soc. Sci. Fish. 47: 605-611.

13. Miki,W., K. Yamaguchi, S. Konosu, and T. Watanabe. 1984. Metabolism of dietary carotenoids in eggs of red sea bream. Comp. Biochem. Physiol. 77: 665 -668.

14. Misawa, N., Y. Satomi, K. Kondo, A. Yokoyama, S. Kajiwara, T. Saito, T. Ohtani, and W. Miki. 1995. Structure and functional analysis of a marine bacterial carotenoid biosynthesis gene cluster and astaxanthin biosynthetic pathway proposed at the gene level. J Bacteriol. 177: 6575-6584.

15. Schiedt, K., F. J. Leuenberger, M. Vecchi, and E. Glinz. 1985. Absorption, retention and metabolic transformations of carotenoids in rainbow trout, salmon and chicken. Pure Appl. Chem. 57: 685-692.

16. Schiedt, K., M. Vecchi, E. Glinz, and T. Storebakken. 1988. Metabolism of carotenoids in salmonids: metabolism of astaxanthin and canthaxanthin in the skin of atlantic salmon (*Salmo salar, L.*). Helv. Chim. Acta 71:887-896.

17. Stradi, R., G. Celentano, E. Rossi, G. Rovati, and M. Pastore. 1995. Carotenoids in bird plumage: I. The carotenoid pattern in a series of Palearctic Carduelinae. Comp. Biochem. Physiol. 110:131 -143.

18. Tsushima, M., T. Kawakami, and T. Matsuno. 1993. Metabolism of carotenoids in sea-urchin *Pseudocentrotus depressus*. Comp. Biochem. Physiol. 106: 737 -741.

19. Wyss, A., G. Wirtz, W. Woggon, R. Brugger, M. Wyss, A. Friedlein, H. Bachmann, and W. Hunziker. 2000. Cloning and expression of beta,beta-carotene 15,15'-dioxygenase. Biochem Biophys Res Commun. 271:334-336.

20. Liu, B.-H. and Y.-K. Lee 1999. Composition and biosynthetic pathways of carotenoids in the astaxanthin-producing green alga *Chlorococcum sp.* Biotechnology Letters 21(11): 1007-1010.

21. Fraser, P. D. and P. M. Bramley (2004). The biosynthesis and nutritional uses of carotenoids. Progress in Lipid Research 43(3): 228-265.

22. Martin, J., E. Gudina, et al. 2008. Conversion of beta-carotene into astaxanthin: two separate enzymes or a bifunctional hydroxylase-ketolase protein? Microbial Cell Factories 7(1): 3.

23. Punginelli, C., A. Wilson, et al. 2009. Influence of zeaxanthin and echinenone binding on the activity of the orange carotenoid protein. Biochimica et Biophysica Acta - Bioenergetics 1787(4): 280-288.

24. Aas, G. H., B. Bjerkeng, et al. 1997. Idoxanthin, a major carotenoid in the flesh of Arctic charr (*Salvelinus alpinus*) fed diets containing astaxanthin. Aquaculture 150(1-2): 135-142.

25. Schwartzel E.M. and J.J Cooney. 1970 Isolation and identification of echinenone from *Micrococcus roseus*. J Bacteriology. 104: 272-274.

26. Esteban, R., B. Martínez, et al. 2009. Carotenoid composition in Rhodophyta: insights into xanthophyll regulation in *Corallina elongate*. European Journal of Phycology 44(2): 221 - 230.

27. Bjerkeng B., Hatlen B. and M. Jobling 2000 Astaxanthin and its metabolites idoxanthin and crustaxanthinin flesh, skin, and gonads of sexually immature and maturingArctic charr (*Salvelinus alpinus* (L.)). Comp Biochem Physiol 99(3): 395-404.

28. Maoka, T. and T. Matsuno 1989. Metabolism of carotenoids in terrestrial snail Euhadra Callizona amaliae. Comparative Biochemistry and Physiology Part B: Comparative Biochemistry 92(1): 41-43.

29. Schiedt, K., Bischof, S. and Glinz, E. 1991. Recent progress on carotenoid metabolism in animals. Pure Appl. Chem. 63: 89-100

30. Matsuno, T., K. Katagiri, et al. 1985. Novel reductive metabolic pathways of 4-oxo-[beta]-end group in carotenoids of the spindle shell *Fusinus perplexus*. Comparative Biochemistry and Physiology Part B: Comparative Biochemistry 81(4): 905-908.

31. Henmi, H., M. Hata, et al. 1991. Studies on the carotenoids in the muscle of salmon--V. Combination of astaxanthin and canthaxanthin with bovine serum albumin and egg albumin. Comparative Biochemistry and Physiology Part B: Comparative Biochemistry 99(3): 609-612.

32. Stradi, R., J. Hudon, et al. 1998. Carotenoids in bird plumage: the complement of yellow and red pigments in true woodpeckers (Picinae). Comparative Biochemistry and Physiology Part B: Biochemistry and Molecular Biology 120(2): 223-230.

33. Krinsky N.I., Landrum J.T. and R.A. Bone. 2003. Biologic mechanisms of the protective role of lutein and zeaxanthin in the eye. Annu. Rev. Nutr. 23:171–201

34. Khachik F. 2006. Distribution and metabolism of dietary carotenoids in humans as a criterion for development of nutritional supplements. Pure Appl. Chem. 78(8): 1551–1557.

35. Goodwin, T. W. 1986. Metabolism, Nutrition, and Function of Carotenoids. Annual Review of Nutrition 6(1): 273-297.

36. Hudon J., Anciães M., Bertacche V. and R. Stradi 2007. Plumage carotenoids of the Pin-tailed Manakin (*Ilicura militaris*): evidence for the endogenous production of rhodoxanthin from a colour variant. Comp Biochem and Physiol Part B 147: 402–411.

37. McGraw, K. J., E. Adkins-Regan, Parker R.S. 2002. Anhydrolutein in the zebra finch: a new, metabolically derived carotenoid in birds. Comparative Biochemistry and Physiology Part B: Biochemistry and Molecular Biology 132(4): 811-818.

38. McGraw K.J., Hill G.E., Stradi R., Parker R.S. 2002. The effect of dietary carotenoid access on sexual dichromatism and plumage pigment composition in the American goldfinch. Comparative Biochemistry and Physiology *B* 131: 261–269.

39. Matsuno, T. 2001. Aquatic animal carotenoids. Fisheries Science 67(5): 771-783.

40. Tsushima, M., Y. Ikuno, Nagata S., Kodama K. and T. Matsuno. 2002 Comparative biochemical studies of carotenoids in catfishes. Comparative Biochemistry and Physiology Part B: Biochemistry and Molecular Biology 133(3): 331-336.

41. McGraw, K. J. and M.C. Nogare 2004. Carotenoid pigments and the selectivity of psittacofulvin-based coloration systems in parrots. Comp Biochem Physiol Part B: Biochemistry and Molecular Biology 138(3): 229-233.

42. Ohkubo, M., M. Tsushima, et al. 1999. Carotenoids and their metabolism in the goldfish *Carassius auratus* (Hibuna). Comparative Biochemistry and Physiology Part B: Biochemistry and Molecular Biology 124(3): 333-340.

43. Andersson, Staffan, et al. 2007. Carotenoid content and reflectance of yellow and red nuptial plumages in widowbirds (*Euplectes* spp.). Functional Ecology 21:272-281.

44. Maoka, T., A. Arai, et al. 1986. The first isolation of enantiomeric and meso-zeaxanthin in nature. Comparative Biochemistry and Physiology Part B: Comparative Biochemistry 83(1): 121-124.

45. Stradi, R., E. Pini, et al. 2001. Carotenoids in bird plumage: the complement of red pigments in the plumage of wild and captive bullfinch (*Pyrrhula pyrrhula*). Comparative Biochemistry and Physiology Part B: Biochemistry and Molecular Biology 128(3): 529-535.

46. Bhosale, P., B. Serban, et al. 2007. Identification and Metabolic Transformations of Carotenoids in Ocular Tissues of the Japanese Quail *Coturnix japonica* Biochemistry 46(31): 9050-9057.

47. Siefermann-Harms, D., Hertzberg, S., Borch, G. and S. Liaaen-Jensen. 1981 Lactucaxanthin, an ε,ε-carotene-3,3′-diol from *Lactuca sativa*. Phytochemistry 20:85–88.

48. Bai, L., E. H. Kim, et al. 2009. Novel lycopene epsilon cyclase activities in maize revealed through perturbation of carotenoid biosynthesis. The Plant Journal 59(4): 588-599.

49. Asai, A., M. Terasaki, et al. 2004. An epoxide-furanoid rearrangement of spinach neoxanthin occurs in the gastrointestinal tract of mice and in vitro: formation and cytostatic activity of neochrome stereoisomers. J. Nutr. 134(9): 2237-2243.

50. Hallenstvet, M., E. Ryberg, et al. 1978. Animal carotenoids--XIV carotenoids of *Psammechinus miliaris* (sea-urchin). Comparative Biochemistry and Physiology Part B: Comparative Biochemistry 60(2): 173-175.

51. Yonekura, L., M. Kobayashi, Terasaki M., and A. Nagao. 2010 Keto-carotenoids are the major metabolites of dietary lutein and fucoxanthin in mouse tissues. J. Nutr. 140: 1824-1831

52. Tsushima, M. and T. Matsuno 1990. Comparative biochemical studies of carotenoids in sea-urchins-I. Comparative Biochemistry and Physiology Part B: Comparative Biochemistry 96(4): 801-810.

53. Fox, D. L. and T. S. Hopkins 1966. Comparative metabolic fractionation of carotenoids in three flamingo species. Comparative Biochemistry and Physiology 17(3): 841-856.

54. Toyoda, Y., L. R. Thomson, et al. 2002. Effect of Dietary Zeaxanthin on Tissue Distribution of Zeaxanthin and Lutein in Quail. Invest. Ophthalmol. Vis. Sci. 43(4): 1210-1221.

55. Arpin, N. and S. Liaaen-Jensen 1969. Carotenoids of higher plants--II: Rubixanthin and gazaniaxanthin. Phytochemistry 8(1): 185-193.

56. Deviche, P., K. J. McGraw, et al. 2008. Season-, sex-, and age-specific accumulation of plasma carotenoid pigments in free-ranging white-winged crossbills Loxia leucoptera. Journal of Avian Biology 39(3): 283-292.

57. Inouye C.Y., G. E. Hill, Stradi R.D. and R. Montgomerie 2001. Carotenoid pigments in male house finch plumage in relation to age, subspecies, and ornamental coloration. Auk 118(4): 900-915.

58. Valadon, L. R. G. and R. S. Mummery 1969. Changes in Carotenoid Composition of Certain Roses with Age. Annals of Botany 33(4): 671-677.

59. Umeno, D., A. V. Tobias, et al. 2005. Diversifying Carotenoid Biosynthetic Pathways by Directed Evolution. Microbiol. Mol. Biol. Rev. 69(1): 51-78.

60. Niklitschek, M., J. Alcaino, et al. 2008. Genomic organization of the structural genes controlling the astaxanthin biosynthesis pathway of Xanthophyllomyces dendrorhous. Biological Research 41: 93-108.

61. Cunningham, F. X., Jr., H. Lee, et al. 2007. Carotenoid Biosynthesis in the Primitive Red Alga Cyanidioschyzon merolae. Eukaryotic Cell 6(3): 533-545.

62. Sandmann, G. 1994. Carotenoid biosynthesis in microorganisms and plants. European Journal of Biochemistry 223(1): 7-24.

63. Ladygin, V. G. 2000. Biosynthesis of Carotenoids in the Chloroplasts of Algae and Higher Plants. Russian Journal of Plant Physiology 47(6): 796-814.

64. Leuenberger, F., Thommen, H . 1970. Keto-carotenoids in the Colorado beetle Leptinotarsa decemlineata . l. Insect Physiol. 16: 1 855-58.

65. Kayser 1977 Conversion of [C^{14}]β-carotene to its 2-hydroxy and 3-hydroxy metabolites by two moth species. Comp Biochem Physiol. 59: 177-181.

66. Katayama T., Kamata T., Shimaya M., Deshimaru O. and Chichester C. O. (1972) The biosynthesis of astaxanthin--VIII. The conversion of labelled β-carotene-15,15'-$^3H_2$ into astaxanthin in prawn, *Penaeus japonicus* Bat3. *Nippon Suisan Gakkaishi,* 38, 1171-1175.

67. KEGG (reaction R)1851, enzyme 1. 14.13.12 9)

68. Maoka T. 2011 Carotenoids in Marine Animals. 9:278-293293. Mar Drug.

69. Heller K.G., Fleischmann P. and Lutz-Röder A. 2000 Carotenoids in the spermatophores of bushcrickets (Orthoptera: Ephippigerinae). Proceed Royal Soc. 267: 1905-1908.

70. Goodwin T.W. 1984 The biochemistry of the carotenoids. Vol. 1. Plants. Chapman and Hall Eds

71. Goodwin T.W. 1984 The biochemistry of the carotenoids. Vol. 2. Animals. Chapman and Hall Eds.

72. Fox D.L., McBeth, J.W., and G. Mackinney. 1970 Some dietary carotenoids and blood-carotenoid levels in flamingos-II. γ-carotene and α-carotene consumed by the American flamingo. Comparative Biochemistry and Physiology 36:253-262.

73. Choi, S.-K., H. Harada, S. Matsuda, and N. Misawa. 2007. Characterization of two β-carotene ketolases, CrtO and CrtW, by complementation analysis in *Escherichia coli*. Applied Microbiology and Biotechnology 75:1335-1341.

74. Cunningham, F. X., and E. Gantt. 1998. Genes and enzymes of carotenoid biosynthesis in plants. Annu Rev Plant Physiol Mol Biol 49:557 - 583.

75. Fraser, P. D., Y. Miura, and N. Misawa. 1997. In vitro characterization of astaxanthin biosynthetic enzymes. Journal of Biological Chemistry 272:6128-6135.

76. Ravanello, M. P., D. Ke, J. Alvarez, B. Huang, and C. K. Shewmaker. 2003. Coordinate expression of multiple bacterial carotenoid genes in canola leading to altered carotenoid production. Metabolic Engineering 5:255-263.

77. Katayama, T., S. Makoto, S. Muneo, and C.O. Chichester. 1973. The biosynthesis of astaxanthin. XII. The conversion of labelled β-carotene-15, 15 $^3H_2$ into body astaxanthin in the lobster, *Panulirus japonicus*. International Journal of Biochemistry 4:223-226.

78. Katayama, T., Y. Kunisaki, M. Shimaya, K. L. Simpson, and C. O. Chichester. 1973a. The biosynthesis of astaxanthin--XIV. The conversion of labelled [beta]-carotene-15,15'-$^3H_2$ into astaxanthin in the crab, *Portunus trituberculatus*. Comparative Biochemistry and Physiology Part B: Comparative Biochemistry 46:269-272.

79. Withers, N. W., R. S. Alberte, R. A. Lewin, J. P. Thornber, G. Britton, and T. W. Goodwin. 1978. Photosynthetic unit size, carotenoids, and chlorophyll-protein composition of prochloron sp., a prokaryotic green alga. Proceedings of the National Academy of Sciences of the United States of America 75:2301-2305.

80. Cunningham, F. X., and E. Gantt. 2005. A study in scarlet: enzymes of ketocarotenoid biosynthesis in the flowers of Adonis aestivalis. The Plant Journal 41:478-492.

81. Hsieh, L. K., T.-C. Lee, C. O. Chichester, and K. L. Simpson. 1974. Biosynthesis of Carotenoids in Brevibacterium sp. KY-4313. Journal of Bacteriology 118:385-393.

82. Matsuno, T., K. Katagiri, T. Maoka, and T. Komori. 1985. Novel reductive metabolic pathways of 4-oxo-[beta]-end group in carotenoids of the spindle shell Fusinus perplexus. Comparative Biochemistry and Physiology Part B: Comparative Biochemistry 81:905-908.

83. Gilchrist, B. M., and W. L. Lee. 1976. The incorporation of [14C] β-carotene into the marine isopod *Idotea resecata* (Stimpson, 1857) and the biosynthesis of canthaxanthin. Comparative Biochemistry and Physiology Part B: Comparative Biochemistry 54:343-346.

84. Yuan, J.-P., and F. Chen. 1997. Identification of astaxanthin isomers in *Haematococcus lacustris* by HPLC-photodiode array detection. Biotechnology Techniques 11:455-459.

85. Schiedt, K., F. J. Leuenberger, and M. Vecchi. 1981. Natural occurrence of enantiomeric and meso-astaxanthin. 5. Ex wild salmon (*Salmo salar* and *Oncorhynchus*). Helvetica Chimica Acta 64:449-457.

86. Toomey, M. B., and K. J. McGraw. 2010. The effects of dietary carotenoid intake on carotenoid accumulation in the retina of a wild bird, the house finch (Carpodacus mexicanus). Archives of Biochemistry and Biophysics 504:161–168.

87. Cunningham, F. X., and E. Gantt. 2001. One ring or two? Determination of ring number in carotenoids by lycopene epsilon-cyclases. Proceedings of the National Academy of Sciences 98:2905-2910.

88. Hora, J., T. P. Toube, and B. C. L. Weedon. 1970. Carotenoids and related compounds. Part XXVII. Conversion of fucoxanthin into paracentrone. Journal of the Chemical Society C: Organic 2:241-242.

89. KEGG (reaction R07568, enzyme CtlZ)

90. Goodfellow D., Moss G. P. and B.C.L. Weedon. 1970. The Absolute Configuration of Lutein. J. Chem. Soc. D 13:1578-1578.

91. Khachik, F., P. S. Bernstein, and D. L. Garland. 1997. Identification of lutein and zeaxanthin oxidation products in human and monkey retinas. Investigative Ophthalmology & Visual Science 38:1802-11.

92. Hata, M., and M. Hata. 1971. Carotenoid pigments in goldfish (carassius auratus) II. colour change and carotenoid pigment composition. International Journal of Biochemistry 2:182-184.

93. Stradi, R., G. Celentano, M. Boles, and F. Mercato. 1997. Carotenoids in bird plumage: The pattern in a series of red-pigmented carduelinae. Comparative Biochemistry and Physiology Part B: Biochemistry and Molecular Biology 117:85-91.

94. Takaichi, S., K. Shimada, and J. Ishidsu. 1990. Carotenoids from the aerobic photosynthetic bacterium, *Erythrobacter longus*: β-Carotene and its hydroxyl derivatives. Archives of Microbiology 153:118-122.

95. Czeczuga, B. 1985. Carotenoids in representatives of the cladoniaceae. Biochemical Systematics and Ecology 13:83-88.

96. Cunningham, F. X. J., and G. E. 2011. Elucidation of the Pathway to Astaxanthin in the Flowers of *Adonis aestivalis*. The Plant Cell 23:3055-3069.

97. Takaichi, S., G. Sandmann, G. Schnurr, Y. Satomi, A. Suzuki, and N. Misawa. 1996. The carotenoid 7, 8-dihydro-ψ end group can be cyclized by the lycopene cyclases from the bacterium *Erwinia Uredovora* and the higher Plant *Capsicum Annuum*. European Journal of Biochemistry 241:291-296.

98. Gribanovski-Sassu, O. 1972. Effect of diphenylamine on carotenoid synthesis in *Dictyococcus cinnabarinus*. Phytochemistry 11:3195-3198.

99. Hudon, J., and A. H. Brush. 1990. Carotenoids produce flush in the elegant tern plumage. The Condor 92:798-801.

100. Schwartzel, E. M., and J. J. Cooney. 1972. Isolation of 4′-Hydroxyechinenone from Micrococcus roseus. Journal of Bacteriology 112: 1422-1424.

101. McGraw, K. J., P. M. Nolan, and O. L. Crino. 2006. Carotenoid accumulation strategies for becoming a colourful house finch: analyses of plasma and liver pigments in wild moulting birds. Functional Ecology 20:678-688.

102. Makino, T., H. Harada, H. Ikenaga, S. Matsuda, S. Takaichi, K. Shindoet al. (2008). Characterization of Cyanobacterial Carotenoid Ketolase CrtW and Hydroxylase CrtR by Complementation Analysis in Escherichia coli. Plant and Cell Physiology 49: 1867-1878.

103. Teruhisa Katayama, Y. K., Makoto Shimaya, K.L. Simpson and C.O. Chichester 1973. The biosynthesis of astaxanthin—XIV. The conversion of labelled β-carotene- 15,15′-3H2 into astaxanthin in the crab, *Portunus trituberculatus*. Comp Biochem 46: 269-272.

104. Stradi, R., E. Rossi, G. Celentano and B. Bellardi. 1996. Carotenoids in bird plumage: The pattern in three Loxia species and in Pinicola enucleator. Comparative Biochemistry and Physiology Part B: Biochemistry and Molecular Biology 113(2): 427-432.

105. Egeland, E. S., G. Johnsen, W. Eikrem, J. Throndsen, et al.1995. Pigments of *Bathycoccus prasinos* (Prasinophyceae): methodological and chemosystematic implications. J. PhycoL 31: 554-561.

106. Fernández, J. A. and J. Burgos.1981. Carotenoid pigments in the flesh and carapace of *Aristaeomorpha foliacea* and *Heterocarpus dorsalis* (crustacea: decapoda). Comparative Biochemistry and Physiology Part B: Comparative Biochemistry 69: 559-575.

107. Miki, W., K. Yamaguchi, S. Konosu, T. Takane, et al.1985. Origin of tunaxanthin in the integument of yellowtail (Seriola quinqueradiata). Comparative Biochemistry 80(2): 195-201.

108. Juola, F.A,. McGraw K. and Dearborn D.C, Carotenoids and throat pouch coloration in the great frigatebird (*Fregata minor*). Biochemistry and Molecular Biology 149:370-377.

109. Hata and Hata 1971. Carotenoid pigments in goldfish (*Carassius auratus* L.)-III. Metabolism of ingested cynthiaxanthin. Tohoku J. Agri. Res., 21 (19716), pp. 183–188

110. Buchecker, R. 1982 A chemist's view of animal carotenoids in: Carotenoid Chemistry and Biochemistry. G. Britton, T.W. and Goodwin (Eds.), Pergamon Press, Oxford (1982), p. 175-193.

111. Britton, G., Liaaen-Jensen, S and H. Pfander. 2004 Carotenoid. Handbook. Birkhäuser, Basel, Switzerland.

112. Czeczuga, B. and R. D. Worthington.1997. Carotenoids in lichens from the States of New Mexico and Texas in the United States of America. Feddes Repertorium 108: 387-399.

113. Goodwin, T. W. 1974. Algal physiology and biochemistry, Blackwell Scientific Publications Ltd.

114. Schiedt, K., S. Bischof and E. Glinz. 1993. Carotenoids. Part B. Metabolism, genetics, and biosynthesis, Harcourt Brace Jovanovich Publishers.

115. Han, Q., K. Shinohara, Y. Kakubari and Y. Mukai.2003. Photoprotective role of rhodoxanthin during cold acclimation in Cryptomeria japonica. Plant, Cell & Environment 26(5): 715-723.

116. Maoka, T., Y. Ito, Fujiwara and K. Hashimoto.1996. Structures and antioxidative activity of retro Carotenoids from the Berries of the Japanese yew, Taxus cuspidata. J. Jap. Oil Chem. Soc. 45: 641-646.

117. Hsu, W.-J., D. B. Rodriguez and C. O. Chichester. 1972. The biosynthesis of astaxanthin. VI. the conversion of [14C]lutein and [14C] β-carotene in goldfish. International Journal of Biochemistry 3: 333-338.

118. Hata, M. and Hata, M. 1972 Carotenoid pigments in goldfish - IV. Carotenoid metabolism, Bull. Jap. Soc. Sci. Fish., 38. 331-338.

119. Matsuno, T. and Katsuyama, M. 1982 Metabolism of zeaxanthin to rhodoxanthin in tilapia. Nippon Suisan Gakkaishi, 48 (1982), pp. 1491–1493.

120. Matsuno, T., Nagata, S., Iwahashi, M, Koike, T., Okada M. 1979. Intensification of color of fancy red carp with

zeaxanthin and myxoxanthophyll, major carotenoid constituents of spirulina. Bull. Jap. Soc. Scient. Fish., 45, pp. 627–632.

121. Guillou, A., Choubert, G., de la Noüe, J. 1992. Comparative accumulations of labelled carotenoids (14C-astaxanthin, 3H-canthaxanthin and 3H-zeaxanthin) and their metabolic conversions in mature female rainbow trout (*Oncorhynchus mykiss*). Comp. Biochem. Physiol. B. 102: 61-65.

122. Strand, A., O. Herstad and S. Liaaen-Jensen. 1998. Fucoxanthin metabolites in egg yolks of laying hens. Comparative Biochemistry and Physiology 119: 963-974.

123. McGraw, K. J., G. E. Hill, R. Stradi and R. S. Parker. 2002. The effect of dietary carotenoid access on sexual dichromatism and plumage pigment composition in the American goldfinch. Comparative Biochemistry and Physiology Part B: Biochemistry and Molecular Biology 131: 261-269.

124. Nitsche, H. (1974). Neoxanthin and fucoxanthinol in *Fucus vesiculosus*. Biochimica et Biophysica Acta (BBA) - General Subjects 338: 572-576.

125. Tian, L., Magallanes-Lundback, M., Musetti, V. and D. DellaPenna 2003 Functional Analysis of β- and ε-ring carotenoid hydroxylases in *Arabidopsis* The Plant Cell 15: 1320-1332.

126. Czeczuga, B. 1981. Carotenoids in fish. XXVIII. Carotenoids in *Micropterus salmoides* (Lalépéde) Centrarchidae. Hydrobiologia 78: 45-98.

127. Schiedt, K., Foss, P., Trond, S. and S. Liaaen-Jensen. 1989. Metabolism of carotenoids in salmonids-I. Idoxanthin, a metabolite of astaxanthin in the flesh of Atlantic salmon (*Salmo salar*, L.) under varying external conditions. Comparative Biochemistry and Physiology 92B: 277-281.

128. Hepperle, S.S., Li, Q. and A.L.L. East. 2005. Mechanism of cis/trans equilibration of alkenes via iodine catalysis. Journal of Physical Chemistry A 109: 10975-10981.

129. McDermott, J.C.B., Brown, D.J., Britton, G. and T.W. Goodwin. 1974. Alternative pathways of zeaxanthin biosynthesis in a *Flavobacterium* species. Experiments with nicotine as inhibitor. Biochemical Journal 144: 231-243.

130. Buchecker, R., Eugster, C.H. 1978. 183. Absolute konfiguration von α-doradexanthin und von frtischiellaxanthin, einem neuen carotenoid aus *Fritschiella tuberose* IYENG. Helvetica Chimica Acta 61: 1962-1968.

131. KEGG (reaction R07850, enzyme 1. 14. 99. 45).

200. Prum, R. O., A. M. LaFountain, J. Berro, M. C. Stoddard, and H. A. Frank. 2012. Molecular diversity, metabolic transformation, and evolution of carotenoid feather pigments in cotingas (Aves: Cotingidae). J Comp Physiol B 182:1095-1116.

201. Prum, R., A. LaFountain, C. Berg, M. Tauber, and H. Frank. 2014. Mechanism of carotenoid coloration in the brightly colored plumages of broadbills (Eurylaimidae). J Comp Physiol B 184:651-672.

202. LaFountain, A. M., H. A. Frank, and R. O. Prum. 2013. Carotenoids from the crimson and maroon plumages of Old World orioles (Oriolidae). Archives of Biochemistry and Biophysics 539:126-132.

203. LaFountain, A. M., S. Kaligotla, S. Cawley, K. M. Riedl, S. J. Schwartz, H. A. Frank, and R. O. Prum. 2010. Novel methoxy-carotenoids from the burgundy-colored plumage of the Pompadour Cotinga Xipholena punicea. Archives of Biochemistry and Biophysics 504:142-153.

Appendix S2. Characteristics of carotenoid metabolic networks for species used in the study. See methods for details on measurements.

Common name	Scientific Name	Diet nodes	Nodes	Edges	Diam	Path	Degree	Diet diam	Cluster	Assort	Heter	Centr	Density	Modularity	Modules	Sensit/edge	Sens/node	Method	Main references
Long Tailed Tit	Aegithalos_caudatus	1	2	1	1	1.00	1.00	1.00	0.00	1.00	0.00	.	1.00	0.00	1	1.00	0.75	HPLC	62, 63
Red-winged Blackbird	Agelaius_phoeniceus	4	17	37	6	2.47	4.35	4.00	0.41	3.33	0.37	0.15	0.18	0.53	4	0.00	0.06	HPLC	11, 38, 47
Red-legged Partridge	Alectoris_rufa	3	17	31	6	2.62	3.65	4.00	0.36	2.66	0.32	0.12	0.15	0.61	3			HPLC	100, 101
Red Munia	Amandava_amandava	3	5	4	3	1.57	1.60	1.00	0.00	1.75	0.62	0.33	0.30	0.17	3	0.13	0.24	HPLC	40
Zebra Waxbill	Amandava_subflava	3	5	4	3	1.57	1.60	1.00	0.00	1.75	0.62	0.33	0.30	0.17	3	0.13	0.24	HPLC	40
Scaled fruiteater	Ampelioides_tschudii	1	1	0	0	0.00	0.00	0.00	0.00	.	.	.	0.00	0.00	1			HPLC	103
Chestnut-crested cotinga	Ampelion_rufaxilla	1	1	0	0	0.00	0.00	0.00	0.00	.	.	.	0.00	0.00	1			HPLC	103
Mallard	Anas_platyrhynchos	6	8	6	2	1.25	1.50	2.00	0.00	1.67	0.71	0.19	0.14	0.50	4	0.08	0.13	HPLC2	9, 12, 53
Greylag Goose	Anser_anser	3	3	0	0	0.00	0.00	0.00	0.00	.	.	.	0.00	0.00	3	.	0.33	HPLC2	12, 50
Helmeted manakin	Antilophia_galeata	2	7	11	4	2.00	3.00	4.00	0.32	2.82	0.35	0.29	0.36	0.36	2			HPLC	105
Narina trogon	Apaloderma_narina	4	16	32	8	3.06	3.76	7.00	0.35	3.04	0.46	0.18	0.15	0.53	4			HPLC	111
Cedar Waxwing	Bombycilla_cedrorum	5	13	20	3	1.57	3.08	3.00	0.36	2.99	0.60	0.28	0.18	0.44	5	0.01	0.09	HPLC2	8, 11, 26, 62
Bohemian Waxwing	Bombycilla_garrulus	2	9	18	3	1.39	4.00	3.00	0.53	2.90	0.25	0.21	0.33	0.41	2	0.00	0.19	HPLC	62, 63
Japanese Waxwing	Bombycilla_japonica	2	9	18	3	1.39	4.00	3.00	0.53	2.90	0.25	0.21	0.33	0.41	2	0.00	0.19	HPLC	62
Trumpeter Finch	Bucanetes_githagineus	4	16	32	8	2.91	4.00	6.00	0.37	3.39	0.50	0.26	0.18	0.47	4	0.01	0.07	HPLC	63
Yellow-rumped cacique	Cacicus_cela	1	1	0	0	0.00	0.00	0.00	0.00	.	.	.	0.00	0.00	1			HPLC	108
Red-rumped cacique	Cacicus_haemorrhous	4	17	35	6	2.73	4.12	4.00	0.42	2.84	0.30	0.10	0.16	0.59	4			HPLC	108
Northern mountain cacique	Cacicus_leucoramphus	2	2	0	0	0.00	0.00	0.00	0.00	.	.	.	0.00	0.00	2			HPLC	108
Mexican cacique	Cacicus_melanicterus	2	2	0	0	0.00	0.00	0.00	0.00	.	.	.	0.00	0.00	2			HPLC	108
Scarlet-rumped cacique	Cacicus_uropygialis	3	12	21	5	2.00	3.50	3.00	0.41	2.62	0.32	0.18	0.21	0.55	3			HPLC	108
Green broadbill	Calyptomena_viridis	1	4	2	2	1.33	1.00	2.00	0.00	1.86	0.71	0.67	0.33	0.00	2			HPLC	106
Cream-backed Woodpecker	Campephilus_leucopogon	4	16	32	8	2.91	4.00	6.00	0.37	3.39	0.50	0.26	0.18	0.47	4	0.01	0.07	HPLC	68
Pyrrhuloxia	Cardinalis_sinuatis	4	16	30	6	2.76	3.75	5.00	0.38	2.84	0.44	0.20	0.16	0.54	4			HPLC	111
Northern Cardinal	Cardinalis_cardinalis	5	20	40	9	3.01	4.00	6.00	0.33	3.49	0.48	0.19	0.14	0.51	5	0.01	0.06	HPLC2	11, 28, 43, 46
Black Siskin	Carduelis_atrata	1	4	6	2	1.14	3.00	1.00	0.46	2.67	0.20	0.33	0.83	0.00	1	0.06	0.44	HPLC	62, 63
Linnet	Carduelis_cannabina	6	18	31	6	2.55	3.44	4.00	0.33	2.86	0.61	0.18	0.13	0.51	6	0.01	0.06	HPLC	3, 62, 69
European Goldfinch	Carduelis_carduelis	3	10	18	3	1.39	3.60	3.00	0.48	2.90	0.43	0.22	0.27	0.41	3	0.00	0.16	HPLC2	28, 64, 65
European Greenfinch	Carduelis_chloris	2	5	8	3	1.42	3.20	3.00	0.33	2.73	0.33	0.25	0.60	0.11	2	0.00	0.20	HPLC	54, 60, 64, 65
Red Siskin	Carduelis_cucullata	4	16	32	8	2.91	4.00	6.00	0.37	3.39	0.50	0.26	0.18	0.47	4	0.01	0.07	HPLC	62, 63
Common Redpoll	Carduelis_flammea	5	17	31	6	2.55	3.65	4.00	0.35	2.86	0.54	0.19	0.15	0.51	5	0.01	0.07	HPLC	62, 67, 69
Hoary Redpoll	Carduelis_hornemanni	5	18	33	8	2.90	3.67	6.00	0.33	3.12	0.55	0.24	0.14	0.51	5	0.01	0.07	HPLC	67
Oriental Greenfinch	Carduelis_sinica	1	4	6	2	1.14	3.00	1.00	0.46	2.67	0.20	0.33	0.83	0.00	1	0.06	0.44	HPLC	65
Yellow-breasted Greenfinch	Carduelis_spinoides	1	4	6	2	1.14	3.00	1.00	0.46	2.67	0.20	0.33	0.83	0.00	1	0.06	0.44	HPLC	65
Eurasian Siskin	Carduelis_spinus	1	4	6	2	1.14	3.00	1.00	0.46	2.67	0.20	0.33	0.83	0.00	1	0.06	0.44	HPLC	62, 64, 65
American Goldfinch	Carduelis_tristis	4	7	8	3	1.42	2.29	3.00	0.24	2.73	0.75	0.30	0.29	0.11	4	0.00	0.14	HPLC	11, 48
House Finch	Carpodacus_mexicanus	6	21	46	9	3.04	4.00	8.00	0.27	3.32	0.50	0.17	0.12	0.55	6	0.01	0.06	HPLC2	3, 6, 32, 42, 70, 71
Dark-breasted Rosefinch	Carpodacus_nipalensis	3	12	24	4	1.80	4.00	4.00	0.33	3.08	0.35	0.26	0.24	0.44	3	0.00	0.08	HPLC	63
Beautiful Rosefinch	Carpodacus_pulcherrimus	4	16	25	6	2.34	3.13	6.00	0.18	2.81	0.53	0.29	0.15	0.50	4	0.03	0.11	HPLC	63, 67
Pallas' Rosefinch	Carpodacus_roseus	5	18	26	6	2.33	2.89	6.00	0.16	2.61	0.57	0.26	0.12	0.54	5	0.03	0.09	HPLC	62, 69
Streaked Rosefinch	Carpodacus_rubicilloides	2	4	2	1	1.00	1.00	1.00	0.00	1.00	0.00	0.00	0.33	0.50	2	0.50	0.38	HPLC	63, 67, 69
White-browed Rosefinch	Carpodacus_thura	4	13	19	5	1.91	2.92	3.00	0.23	2.47	0.56	0.30	0.17	0.47	4	0.02	0.10	HPLC	63
Three-banded Rosefinch	Carpodacus_trifasciatus	3	10	17	4	1.88	3.40	4.00	0.30	2.71	0.53	0.39	0.24	0.33	4	0.01	0.11	HPLC	63, 67
Hooded berryeater	Carpornis_cucullatus	1	1	0	0	0.00	0.00	0.00	0.00	.	.	.	0.00	0.00	1			HPLC	103
Swallow-tailed manakin	Chiroxiphia_caudata	2	7	12	4	2.00	3.00	4.00	0.32	2.82	0.35	0.29	0.36	0.36	2			HPLC	105
Blue-backed manakin	Chiroxiphia_pareola	2	7	11	4	2.00	3.00	4.00	0.32	2.82	0.35	0.29	0.36	0.66	2			HPLC	105
Sooty-capped Bush Tanager	Chlorospingus_pileatus	1	1	0	0	0.00	0.00	0.00	0.00	.	.	.	0.00	0.00	1	.	1.00	OTH	35
White Stork	Ciconia_ciconia	2	2	0	0	0.00	0.00	0.00	0.00	.	.	.	0.00	0.00	2	.	0.50	TLC	49
Hooded Grosbeak	Coccothraustes_abeillei	1	1	0	0	0.00	0.00	0.00	0.00	.	.	.	0.00	0.00	1	.	1.00	OTH	38
Evening Grosbeak	Coccothraustes_vespertina	1	1	0	0	0.00	0.00	0.00	0.00	.	.	.	0.00	0.00	1	.	1.00	HPLC	45
Bananaquit	Coereba_flaveola	1	4	6	2	1.14	3.00	1.00	0.46	2.67	0.20	0.33	0.83	0.00	1	0.06	0.44	TLC	30
Yellow-shafted Flicker	Colaptes_auratus	4	18	34	8	2.88	3.78	6.00	0.33	3.23	0.49	0.23	0.15	0.52	4	0.01	0.07	HPLC	11, 62, 68
Red-shafted Flicker	Colaptes_auratus_cafer	4	16	32	8	2.91	4.00	6.00	0.37	3.39	0.50	0.26	0.18	0.47	4	0.01	0.07	HPLC	62
Campo Flicker	Colaptes_campestris	3	3	0	0	0.00	0.00	0.00	0.00	.	.	.	0.00	0.00	3	.	0.33	HPLC	68
Gilded Flicker	Colaptes_chrysoides	3	11	19	6	2.29	3.45	5.00	0.27	3.15	0.55	0.44	0.24	0.35	3	0.02	0.12	HPLC	62
Green-barred Woodpecker	Colaptes_melanochloros	3	13	21	6	2.24	3.23	5.00	0.23	2.97	0.52	0.36	0.19	0.42	3	0.03	0.11	HPLC	62, 68
Lovely cotinga	Cotinga_amabilis	1	5	10	6	2.52	3.11	6.00	0.34	2.49	0.35	0.29	0.28	0.36	3			HPLC	103
Purple-breasted cotinga	Cotinga_cotinga	1	5	10	6	2.52	3.11	6.00	0.34	2.49	0.35	0.29	0.28	0.36	3			HPLC	103
Banded cotinga	Cotinga_maculata	1	5	10	6	2.52	3.11	6.00	0.34	2.49	0.35	0.29	0.28	0.36	3			HPLC	103
Blue Tit	Cyanistes_caeruleus	2	2	0	0	0.00	0.00	0.00	0.00	.	.	.	0.00	0.00	2	.	0.50	HPLC	2, 62
Black-and-red broadbill	Cymbirhynchus_macrorhynchos	1	4	3	4	2.00	1.60	4.00	0.00	1.80	0.31	0.17	0.40	0.22	2			HPLC	106
Great Spotted Woodpecker	Dendrocopos_major	3	11	19	6	2.29	3.45	5.00	0.27	3.15	0.55	0.44	0.24	0.35	3	0.02	0.12	HPLC	62, 68
Yellow-rumped Warbler	Dendroica_coronata	1	1	0	0	0.00	0.00	0.00	0.00	.	.	.	0.00	0.00	1	.	1.00	OTH	38
Palm Warbler	Dendroica_palmarum	1	1	0	0	0.00	0.00	0.00	0.00	.	.	.	0.00	0.00	1	.	1.00	OTH	38
Yellow Warbler	Dendroica_petechia	4	4	0	0	0.00	0.00	0.00	0.00	.	.	.	0.00	0.00	4	.	0.25	HPLC	45
Pileated Woodpecker	Dryocopus_pileatus	3	15	23	6	2.20	3.07	5.00	0.15	2.84	0.52	0.31	0.16	0.45	3	0.03	0.11	HPLC	68
Yellowhammer	Emberiza_citrinella	2	2	0	0	0.00	0.00	0.00	0.00	.	.	.	0.00	0.00	2	.	0.50	HPLC	62, 63
Black-headed Bunting	Emberiza_melanocephala	2	2	0	0	0.00	0.00	0.00	0.00	.	.	.	0.00	0.00	2	.	0.50	HPLC	62, 63

Robin	Erithacus_rubecula	1	2	1	1	1.00	1.00	1.00	0.00	1.00	0.00	.	1.00	0.00	1	1.00	0.75	HPLC	63
Gouldian Finch	Erythrura_gouldiae	2	12	21	5	2.40	3.50	4.00	0.25	2.84	0.35	0.27	0.23	0.40	3	0.04	0.16	HPLC	63
Red-headed Parrot Finch	Erythrura_psittacea	2	8	13	4	1.90	3.25	4.00	0.46	2.88	0.48	0.52	0.32	0.29	3	0.04	0.22	HPLC	63
Scarlet Ibis	Eudocimus_ruber	1	1	0	0	0.00	0.00	0.00	0.00	.	.	.	0.00	0.00	1	.	1.00	TLC	21
Thick-billed Euphonia	Euphonia_laniirostris	4	7	5	2	1.33		3.00	0.00	2.50	1.00	0.60	0.20	0.00	1			HPLC	11, 111
Orange-crowned euphonia	Euphonia_saturata	2	5	5	2	1.33		3.00	0.00	1.67	0.50	0.58	0.40	0.22	2			HPLC	111
Yellow-crowned Bishop	Euplectes_afer	2	3	3	2	1.25	2.00	1.00	0.00	1.67	0.35	1.00	0.67	0.00	1	0.00	0.33	HPLC	55, 63
Red-collared Widowbird	Euplectes_ardens	4	19	40	9	3.01	4.21	6.00	0.35	3.49	0.42	0.20	0.16	0.51	3	0.01	0.06	HPLC	1
Fan-tailed Widowbird	Euplectes_axillaris	2	6	9	3	1.53	3.00	3.00	0.24	3.07	0.47	0.50	0.47	0.12	2	0.03	0.19	HPLC	1
Yellow Bishop	Euplectes_capensis	2	2	0	0	0.00	0.00	0.00	0.00	.	.	.	0.00	0.00	2	.	0.50	HPLC	63
Yellow-mantled Widowbird	Euplectes_macrourus	2	5	8	3	1.42	3.20	3.00	0.33	2.73	0.33	0.25	0.60	0.11	2	0.04	0.20	HPLC	1
Southern Red Bishop	Euplectes_orix	4	17	35	9	3.08	4.12	6.00	0.31	3.36	0.45	0.23	0.17	0.47	4	0.01	0.07	HPLC	55, 63
Banded broadbill	Eurylaimus_javanicus	1	6	5	4	1.73	2.00	4.00	0.00	2.13	0.25	0.19	0.29	0.37	2			HPLC	106
Black-and-yellow broadbill	Eurylaimus_ochromalus	1	6	5	4	1.73	2.00	4.00	0.00	2.13	0.25	0.19	0.29	0.37	2			HPLC	106
Wattled broadbill	Eurylaimus_steerii	1	4	3	4	2.00	1.60	4.00	0.00	1.80	0.31	0.17	0.40	0.22	2			HPLC	106
Korean Flycatcher	Ficedula_zanthopygia	2	2	0	0	0.00	0.00	0.00	0.00	.	.	.	0.00	0.00	2	.	1.00	HPLC	63
Red Fody	Foudia_madagascariensis	4	16	32	8	2.91	4.00	6.00	0.37	3.39	0.50	0.26	0.18	0.47	4	0.01	0.07	HPLC	63
Great Frigatebird	Fregata_minor	4	5	4	2	1.20	1.60	2.00	0.00	1.40	0.33	0.33	0.30	0.44	2	0.50	0.20	HPLC	36
Chaffinch	Fringilla_coelebs	4	12	20	4	1.83	3.33	4.00	0.19	2.61	0.53	0.31	0.20	0.39	4	0.01	0.09	HPLC	62
Brambling	Fringilla_montifringilla	1	1	0	0	0.00	0.00	0.00	0.00	.	.	.	0.00	0.00	1	.	1.00	OTH	38
Domestic Chicken	Gallus_gallus_domesticus	4	15	34	6	2.61	4.53	5.00	0.37	3.53	0.47	0.18	0.20	0.44	4	0.00	0.07	HPLC2	12, 72
Common Yellowthroat	Geothlypis_trichas	4	4	0	0	0.00	0.00	0.00	0.00	.	.	.	0.00	0.00	4	.	0.25	HPLC	11, 45
Crimson fruitcrow	Haematoderus_militaris	3	11	11	5	2.05		5.00	0.10	1.93	0.43	0.11	0.11	0.67	4			HPLC	103
Scarlet Finch	Haematospiza_sipahi	5	18	33	8	2.90	3.67	6.00	0.33	3.12	0.55	0.24	0.14	0.51	5	0.01	0.07	HPLC	63
Flame-crested Manakin	Heterocercus_linteatus	3	11	19	2	1.25	1.50	6.00	0.00	1.50	0.35	0.14	0.18	0.35	3			HPLC	105
Yellow-breasted Chat	Icteria_virens	1	1	0	0	0.00	0.00	0.00	0.00	.	.	.	0.00	0.00	1	.	1.00	HPLC	37
Bullock's oriole	Icterus_bullockii	2	9	16	2	1.30	3.56	2.00	0.62	2.74	0.28	0.25	0.31	0.50	2			HPLC	107
Orange-backed troupial	Icterus_croconotus	1	3	1	1	1.00		1.00	2.00	1.00	0.33	0.50	0.71	0.00	2			HPLC	107
Hooded oriole	Icterus_cucullatus	1	1	0	0	0.00	0.00	0.00	0.00	.	.	.	0.00	0.00	1			HPLC	107
Hispaniolan oriole	Icterus_dominicensis	1	1	0	0	0.00	0.00	0.00	0.00	.	.	.	0.00	0.00	1			HPLC	107
Audobon's oriole	Icterus_graduacauda	1	1	0	0	0.00	0.00	0.00	0.00	.	.	.	0.00	0.00	1			HPLC	107
Altamira oriole	Icterus_gularis	4	13	24	5	1.85	3.69	3.00	0.36	2.98	0.41	0.25	0.21	0.51	3			HPLC	107
Northern Oriole	Icterus_galbula	3	18	39	9	3.01	4.33	7.00	0.37	3.48	0.38	0.21	0.17	0.51	3	0.01	0.06	TLC	28
Venezuelan troupial	Icterus_icterus	2	5	8	3	1.42	3.20	3.00	0.33	2.73	0.33	0.25	0.60	0.11	2			HPLC	111
Yellow-tailed oriole	Icterus_mesomelas	1	1	0	0	0.00	0.00	0.00	0.00	.	.	.	0.00	0.00	1			HPLC	107
Yellow oriole	Icterus_nigrogularis	1	1	0	0	0.00	0.00	0.00	0.00	.	.	.	0.00	0.00	1			HPLC	107
Spot-breasted oriole	Icterus_pectoralis	1	1	0	0	0.00	0.00	0.00	0.00	.	.	.	0.00	0.00	1			HPLC	107
Black-cowled oriole	Icterus_prosthemelas	1	1	0	0	0.00	0.00	0.00	0.00	.	.	.	0.00	0.00	1			HPLC	107
Streaked-back oriole	Icterus_pustulatus	4	13	24	5	1.85	3.69	3.00	0.36	2.98	0.41	0.25	0.21	0.51	3			HPLC	107
Pin-tailed Manakin	Ilicura_militaris	3	9	12	4	2.00	2.67	4.00	0.29	2.82	0.51	0.29	0.28	0.36	3	0.06	0.17	HPLC	31
White-browed purpletuft	Iodopleura_isabellae	1	5	10	1	1.38		2.00	0.75	2.80	0.33	0.67	0.60	0.11	2			HPLC	102
Ring-billed Gull	Larus_delawarensis	1	1	0	0	0.00	0.00	0.00	0.00	.	.	.	0.00	0.00	1	.	1.00	HPLC	39
Yellow-legged Gull	Larus_michahellis	6	10	13	5	2.21	2.60	4.00	0.00	1.89	0.42	0.06	0.18	0.41	4	0.00	0.10	HPLC	52, 59
Silver-eared Mesia	Leiothrix_argentauris	2	9	16	5	2.21	3.56	4.00	0.33	2.91	0.39	0.41	0.31	0.29	3	0.03	0.19	HPLC	63
Red-billed Leiothrix	Leiothrix_lutea	2	9	16	5	2.21	3.56	4.00	0.33	2.91	0.39	0.41	0.31	0.29	3	0.03	0.22	HPLC	62, 63
Blue-crowned manakin	Lepidothrix_coronata	2	5	8	3	1.42	3.20	2.00	0.33	2.73	0.33	0.25	0.60	0.11	2			HPLC	105
Snow-capped manakin	Lepidothrix_nattereri	2	5	8	3	1.42	3.20	2.00	0.33	2.73	0.33	0.25	0.60	0.11	2			HPLC	105
White-fronted manakin	Lepidothrix_serena	2	5	7	3	1.42	3.20	2.00	0.33	2.73	0.33	0.25	0.60	0.11	2			HPLC	105
Franklin's Gull	Leucophaeus_pipixcan	1	1	0	0	0.00	0.00	0.00	0.00	.	.	.	0.00	0.00	1	.	1.00	HPLC	39
Rose-collared piha	Lipaugus_streptophorus	1	5	10	6	2.52	3.11	6.00	0.34	2.49	0.35	0.29	0.28	0.36	3			HPLC	103
Red Crossbill	Loxia_curvirostra	4	10	9	2	1.10	1.80	1.00	0.18	1.67	0.50	0.19	0.18	0.56	4	0.07	0.16	HPLC	13, 62, 64, 66, 69
White-winged Crossbill	Loxia_leucoptera	6	21	39	6	2.45	3.71	4.00	0.33	2.88	0.48	0.14	0.12	0.58	6	0.00	0.05	HPLC2	14, 28, 62, 66
Siberian Rubythroat	Luscinia_calliope	4	16	32	8	2.91	4.00	6.00	0.37	3.39	0.50	0.26	0.18	0.47	4	0.01	0.07	HPLC	62, 63
Eastern striped manakin	Machaeropterus_regulus	2	7	12	4	2.00	3.00	4.00	0.32	2.82	0.35	0.29	0.36	0.36	2			HPLC	105
Red-backed Fairy-Wren	Malurus_melanocephalus	4	19	40	9	3.01	4.21	6.00	0.35	3.49	0.42	0.20	0.16	0.51	4	0.01	0.06	HPLC	57
Golden-winged Manakin	Masius_chrysopterus	2	6	9	3	1.59	3.00	3.00	0.24	3.07	0.47	0.50	0.47	0.12	2	0.03	0.20	HPLC	31
Acorn woodpecker	Melanerpes_formicivorus	2	6	5	2	1.29	1.67	2.00	0.00	1.67	0.35	0.20	0.27	0.50	2			HPLC	111
White Woodpecker	Melanerpes_candidus	2	6	4	2	1.33	1.64	2.00	0.00	1.97	0.35	0.20	0.27	0.50	2	0.38	0.33	HPLC	68
Lewis's Woodpecker	Melanerpes_lewis	4	16	32	8	2.91	4.00	6.00	0.37	3.39	0.50	0.26	0.18	0.47	4	0.01	0.07	HPLC	68
Wild Turkey	Meleagris_gallopavo	4	9	12	3	1.64	2.67	2.00	0.41	3.00	0.79	0.52	0.22	0.21	5	0.02	0.15	TLC	12
Yellow Wagtail	Motacilla_flava	2	2	0	0	0.00	0.00	0.00	0.00	.	.	.	0.00	0.00	2	.	0.50	HPLC	63
Collared Grosbeak	Mycerobas_affinis	2	2	0	0	0.00	0.00	0.00	0.00	.	.	.	0.00	0.00	2	.	0.50	HPLC	63
White-winged Grosbeak	Mycerobas_carnipes	1	1	0	0	0.00	0.00	0.00	0.00	.	.	.	0.00	0.00	1	.	1.00	OTH	38
Black-and-yellow Grosbeak	Mycerobas_icteroides	1	1	0	0	0.00	0.00	0.00	0.00	.	.	.	0.00	0.00	1	.	1.00	OTH	38
Spot-winged Grosbeak	Mycerobas_melanozanthos	1	1	0	0	0.00	0.00	0.00	0.00	.	.	.	0.00	0.00	1	.	1.00	OTH	38
Star Finch	Neochmia_ruficauda	2	2	0	0	0.00	0.00	0.00	0.00	.	.	.	0.00	0.00	1	.	0.50	HPLC	40
Pale-bellied tyrant-manakin	Neopelma_pallescens	2	5	8	3	1.42	3.20	2.00	0.33	2.73	0.33	0.25	0.60	0.11	2			HPLC	105
Egyptian Vulture	Neophron_percnopterus	1	1	0	0	0.00	0.00	0.00	0.00	.	.	.	0.00	0.00	1	.	1.00	HPLC	51
Tristan Bunting	Nesospiza_acunhae	2	6	9	3	1.53	3.00	3.00	0.24	3.07	0.47	0.50	0.47	0.12	2	0.03	0.19	TLC	58
Hihi (Stitchbird)	Notiomystis_cincta	4	6	4	3	1.57	1.33	1.00	0.00	1.75	0.82	0.30	0.20	0.17	4	0.13	0.19	HPLC	17, 18

Black-and-crimson oriole	Oriolus_cruentus	4	19	35	8	3.17	3.68	6.00	0.32	2.81	0.39	0.16	0.14	0.60	4			HPLC	112
Golden Oriole	Oriolus_oriolus	2	2	0	0	0.00	0.00	0.00	0.00	.	.	.	0.00	0.00	2	.	0.50	HPLC	63
Maroon oriole	Oriolus_traillii	2	10	16	2	1.35	3.09	2.00	0.43	2.58	0.38	0.22	0.22	0.51	3			HPLC	112
Black-hooded Oriole	Oriolus_xanthornus	2	2	0	0	0.00	0.00	0.00	0.00	.	.	.	0.00	0.00	2	.	0.50	HPLC	63
Red-crested cardinal	Paroaria_coronata	4	17	31	8	2.99	3.65	7.00	0.35	2.92	0.37	0.18	0.15	0.56	3			HPLC	111
Great Tit	Parus_major	2	3	1	1	1.00	0.67	1.00	0.00	1.00	0.71	0.50	0.33	0.00	2	1.00	0.44	HPLC3	15, 25, 33, 34, 56, 61, 62
Yellow-cheeked Tit	Parus_spilonotus	2	2	0	0	0.00	0.00	0.00	0.00	.	.	.	0.00	0.00	2	.	0.50	HPLC	63
Grey Partridge	Perdix_perdix	2	8	13	4	1.97	3.25	3.00	0.00	2.81	0.56	0.38	0.29	0.15	4	0.04	0.31	TLC	12
Scarlet Minivet	Pericrocotus_flammeus	4	16	32	8	2.91	4.00	6.00	0.37	3.39	0.50	0.26	0.18	0.47	4	0.01	0.07	HPLC	62
Coal Tit	Periparus_ater	2	2	0	0	0.00	0.00	0.00	0.00	.	.	.	0.00	0.00	2	.	0.50	HPLC	62
Red-tailed tropicbird	Phaethon_rubricauda	3	14	27	8	3.09	3.86	6.00	0.43	2.90	0.40	0.23	0.19	0.50	3			HPLC	111
Ring-necked Pheasant	Phasianus_colchicus	2	8	13	4	1.97	3.25	3.00	0.00	2.81	0.56	0.38	0.29	0.15	4	0.04	0.34	TLC	4, 12
Rose-breasted Grosbeak	Pheucticus_ludovicianus	4	14	29	6	2.58	4.29	4.00	0.43	3.44	0.52	0.22	0.20	0.43	4	0.00	0.07	HPLC2	11, 28
Swallow-tailed cotinga	Phibalura_flavirostris	1	1	0	0	0.00	0.00	0.00	0.00	.	.	.	0.00	0.00	1			HPLC	103
Guianan red cotinga	Phoenicircus_carnifex	2	2	0	3	1.78	3.14	0.00	0.42	2.83	0.28	0.33	0.43	0.00	2			HPLC	103
Andean Flamingo	Phoenicopterus_andinus	3	13	23	4	1.89	3.54	4.00	0.37	3.02	0.49	0.27	0.19	0.50	4	0.01	0.17	TLC	20
Chilean Flamingo	Phoenicopterus_chilensis	1	8	17	4	2.00	4.25	4.00	0.37	3.19	0.45	0.48	0.36	0.25	3	0.01	0.33	TLC	22
James's Flamingo	Phoenicopterus_jamesi	3	13	23	4	1.89	3.54	4.00	0.37	3.02	0.49	0.27	0.19	0.50	4	0.01	0.17	TLC	20
Lesser Flamingo	Phoenicopterus_minor	1	8	17	4	2.00	4.25	4.00	0.37	3.19	0.45	0.48	0.36	0.25	3	0.01	0.33	TLC	22
Greater Flamingo	Phoenicopterus_roseus	1	8	17	4	2.00	4.25	4.00	0.37	3.19	0.45	0.48	0.36	0.25	3	0.01	0.33	TLC	22
American Flamingo	Phoenicopterus_ruber	2	15	30	6	2.50	4.00	5.00	0.40	3.05	0.45	0.20	0.18	0.48	4	0.01	0.08	TLC	20, 24
Three-toed Woodpecker	Picoides_tridactylus	2	2	0	0	0.00	0.00	0.00	0.00	.	.	.	0.00	0.00	2	.	0.50	HPLC	68
Hairy Woodpecker	Picoides_villosus	4	20	36	8	2.85	3.60	6.00	0.26	3.11	0.50	0.21	0.13	0.52	4	0.02	0.07	HPLC	68
Golden-spangled piculet	Picumnus_exilis	2	6	5	2	1.29	1.67	2.00	0.00	1.96	0.35	0.20	0.27	0.50	2			HPLC	111
Scaly-bellied Woodpecker	Picus_squamatus	1	3	2	2	1.33	1.33	2.00	0.00	1.67	0.35	1.00	0.67	0.00	1	0.75	0.67	HPLC	68
Green Woodpecker	Picus_viridis	4	19	35	8	2.87	3.68	6.00	0.31	3.10	0.49	0.22	0.14	0.53	4	0.01	0.07	HPLC	68
Pine Grosbeak	Pinicola_enucleator	4	14	20	5	1.89	2.86	3.00	0.21	2.43	0.54	0.27	0.15	0.51	4	0.03	0.10	HPLC	62, 63, 64, 66, 69
Crimson-hooded manakin	Pipra_aureola	2	7	12	4	2.00	3.00	4.00	0.32	2.82	0.35	0.29	0.36	0.36	2			HPLC	105
Round-tailed Manakin	Pipra_chloromeros	5	17	32	8	2.91	3.76	7.00	0.35	3.39	0.57	0.25	0.15	0.47	5	0.01	0.07	TLC	29
Golden-headed Manakin	Pipra_erythrocephala	4	16	32	8	2.91	4.00	6.00	0.37	3.39	0.50	0.26	0.18	0.47	4	0.01	0.07	TLC	29
Band-tailed manakin	Pipra_fasciicauda	2	7	12	4	2.00	3.00	4.00	0.32	2.82	0.35	0.29	0.36	0.36	2			HPLC	105
Wire-tailed manakin	Pipra_filicauda	2	7	12	4	2.00	3.00	4.00	0.32	2.82	0.35	0.29	0.36	0.36	2			HPLC	105
Red-headed Manakin	Pipra_rubrocapilla	4	16	32	8	2.91	4.00	7.00	0.37	3.39	0.50	0.26	0.18	0.47	4	0.01	0.07	TLC	29
Golden-breasted fruiteater	Pipreola_aureopectus	2	2	0	0	0.00	0.00	0.00	0.00	.	.	.	0.00	0.00	2			HPLC	103
Fiery-throated fruiteater	Pipreola_chlorolepidota	2	2	0	0	0.00	0.00	0.00	0.00	.	.	.	0.00	0.00	2			HPLC	103
Handsome fruiteater	Pipreola_formosa	2	2	0	0	0.00	0.00	0.00	0.00	.	.	.	0.00	0.00	2			HPLC	103
Red-banded fruiteater	Pipreola_whitelyi	1	3	2	4	2.00	3.00	2.00	0.32	2.79	0.35	0.29	0.36	0.00	1			HPLC	103
Hepatic Tanager	Piranga_flava	4	17	37	6	2.47	4.35	4.00	0.41	3.33	0.37	0.15	0.18	0.53	4	0.00	0.06	TLC	28
Western Tanager	Piranga_ludoviciana	2	5	6	2	1.14	2.40	1.00	0.37	2.67	0.55	0.42	0.50	0.00	2	0.06	0.32	TLC	28
Scarlet Tanager	Piranga_olivacea	4	19	40	9	3.01	4.21	6.00	0.35	3.49	0.42	0.20	0.16	0.51	4	0.01	0.06	TLC	11, 28
Summer Tanager	Piranga_rubra	1	5	10	2	1.38	4.00	2.00	0.75	2.80	0.33	0.67	0.60	0.11	2	0.00	0.44	TLC	28
Roseate Spoonbill	Platalea_ajaja	3	3	4	2	1.33	1.33	2.00	0.00	1.67	0.35	1.00	0.67	0.00	1	.	0.33	HPLC2	23, 63
Forest Weaver	Ploceus_bicolor	2	2	0	0	0.00	0.00	0.00	0.00	.	.	.	0.00	0.00	2	.	0.50	HPLC	63
Cape Weaver	Ploceus_capensis	2	2	0	0	0.00	0.00	0.00	0.00	.	.	.	0.00	0.00	2	.	0.50	HPLC	63
Village Weaver	Ploceus_cucullatus	2	2	0	0	0.00	0.00	0.00	0.00	.	.	.	0.00	0.00	2	.	0.50	HPLC	4, 63
Nelicourvi Weaver	Ploceus_nelicourvi	2	2	0	0	0.00	0.00	0.00	0.00	.	.	.	0.00	0.00	2	.	0.50	HPLC	63
Baya Weaver	Ploceus_philippinus	2	2	0	0	0.00	0.00	0.00	0.00	.	.	.	0.00	0.00	2	.	0.50	HPLC	63
Sakalava Weaver	Ploceus_sakalava	2	2	0	0	0.00	0.00	0.00	0.00	.	.	.	0.00	0.00	2	.	0.50	HPLC	63
African-masked Weaver	Ploceus_velatus	2	2	0	0	0.00	0.00	0.00	0.00	.	.	.	0.00	0.00	2	.	0.50	HPLC	63
Purple-throated cotinga	Porphyrolaema_porphyrolaema	1	5	10	6	2.52	3.11	6.00	0.34	2.49	0.28	0.29	0.28	0.36	3			HPLC	103
Three-wattled bellbird	Procnias_tricarunculatus	1	3	4	1	1.00		1.00	0.67	2.00			1.00	0.00	1			HPLC	103
Long-tailed broadbill	Psarisomus_dalhousiae	2	2	0	0	0.00	0.00	0.00	0.00	.	.	.	0.00	0.00	2			HPLC	106
Montezuma oropendola	Psarocolius_montezuma	2	2	0	0	0.00	0.00	0.00	0.00	.	.	.	0.00	0.00	2			HPLC	108
Chestnut-headed oropendola	Psarocolius_wagleri	1	1	0	0	0.00	0.00	0.00	0.00	.	.	.	0.00	0.00	1			HPLC	108
Black-necked aracari	Pteroglossus_aracari	3	11	17	5	2.09	3.09	3.00	0.28	2.31	0.43	0.24	0.20	0.48	3			HPLC	111
Jambu fruit dove	Ptilinopus_jambu	1	1	0	0	0.00	0.00	0.00	0.00	.	.	.	0.00	0.00	1			HPLC	113
Wompoo pigeon	Ptilinopus_magnificus	1	1	0	0	0.00	0.00	0.00	0.00	.	.	.	0.00	0.00	1			HPLC	113
Beautiful fruit dove	Ptilinopus_pulchellus	1	1	0	0	0.00	0.00	0.00	0.00	.	.	.	0.00	0.00	1			HPLC	113
Yellow-bibbed fruit dove	Ptilinopus_solomonensis	1	1	0	0	0.00	0.00	0.00	0.00	.	.	.	0.00	0.00	1			HPLC	113
Red-ruffed fruitcrow	Pyroderus_scutatus	2	10	16	2	1.30	3.20	2.00	0.51	2.50	0.34	0.25	0.24	0.50	2			HPLC	103
Gold-naped Finch	Pyrrhoplectes_epauletta	1	2	1	1	1.00	1.00	1.00	0.00	1.00	0.00	.	1.00	0.00	1	1.00	0.75	OTH	38
Orange Bullfinch	Pyrrhula_aurantiaca	1	4	6	2	1.14	3.00	1.00	0.46	2.67	0.20	0.33	0.83	0.00	1	0.06	0.44	OTH	38
Grey-headed Bullfinch	Pyrrhula_erythaca	3	12	22	5	1.86	3.67	3.00	0.40	2.98	0.38	0.27	0.23	0.50	3	0.01	0.10	HPLC	38, 63
Red-headed Bullfinch	Pyrrhula_erythrocephala	1	4	6	2	1.14	3.00	1.00	0.46	2.67	0.20	0.33	0.83	0.00	1	0.06	0.44	OTH	38
Eurasian Bullfinch	Pyrrhula_pyrrhula	4	19	37	8	2.97	3.89	6.00	0.31	3.29	0.47	0.21	0.15	0.54	4	0.02	0.08	HPLC	62, 69
Cardinal Quelea	Quelea_cardinalis	4	16	32	8	2.91	4.00	6.00	0.37	3.39	0.50	0.26	0.18	0.47	4	0.01	0.07	HPLC	63
Red-headed Quelea	Quelea_erythrops	4	16	32	8	2.91	4.00	6.00	0.37	3.39	0.50	0.26	0.18	0.47	4	0.01	0.07	HPLC	63
Red-billed Quelea	Quelea_quelea	4	16	32	8	2.91	4.00	6.00	0.37	3.39	0.50	0.26	0.18	0.47	4	0.01	0.07	HPLC	63
Purple-throated fruitcrow	Querula_purpurata	3	10	10	6	2.63	3.00	4.00	0.17	2.92	0.58	0.29	0.15	0.67	4			HPLC	103
Toco Toucan	Ramphastos_toco	1	3	2	2	1.33	1.33	2.00	0.00	1.67	0.35	1.00	0.67	0.00	1	0.75	0.67	HPLC	63

White-throated toucan	Ramphastos_tucanus	2	8	9	2	1.18	2.25	2.00	0.17	2.00	0.38	0.24	0.25	0.48	3			HPLC	111
Crimson-backed Tanager	Ramphocelus_dimidiatus	4	14	29	6	2.58	4.14	4.00	0.43	3.44	0.52	0.22	0.20	0.43	4	0.00	0.07	HPLC2	11, 28
Goldcrest	Regulus_regulus	3	11	19	6	2.29	3.45	5.00	0.27	3.15	0.55	0.44	0.24	0.35	3	0.02	0.12	HPLC	62
Golden-crowned Kinglet	Regulus_satrapa	3	18	39	9	3.01	4.33	7.00	0.37	3.48	0.38	0.21	0.17	0.51	3	0.01	0.06	HPLC	10
Desert Finch	Rhodospiza_obsoleta	4	16	32	8	2.91	4.00	6.00	0.37	3.39	0.50	0.26	0.18	0.47	4	0.01	0.07	HPLC	62, 63
Golden-winged Grosbeak	Rhynchostruthus_socotranus	1	4	6	2	1.14	3.00	1.00	0.46	2.67	0.20	0.33	0.83	0.00	1	0.06	0.44	OTH	38
Andean cock-of-the-rock	Rupicola_peruvianus	1	3	4	1	1.00		2.00	0.67	2.00	0.00	0.00	1.00	0.00	2			HPLC	103
Guianan cock-of-the-rock	Rupicola_rupicola	2	10	16	3	1.39	3.09	3.00	0.46	2.45	0.33	0.22	0.22	0.54	3			HPLC	103
Guianan toucanet	Selenidera_piperivora	3	11	17	5	2.09	3.09	3.00	0.28	2.31	0.43	0.24	0.20	0.48	3			HPLC	111
Common Canary	Serinus_canaria	1	4	6	2	1.14	3.00	1.00	0.46	2.67	0.20	0.33	0.83	0.00	1	0.06	0.44	HPLC	4, 63
Citril Finch	Serinus_citrinella	1	4	6	2	1.14	3.00	1.00	0.46	2.67	0.20	0.33	0.83	0.00	1	0.06	0.44	HPLC	62, 65
Yellow-fronted Canary	Serinus_mozambicus	1	4	6	2	1.14	3.00	1.00	0.46	2.67	0.20	0.33	0.83	0.00	1	0.06	0.44	HPLC	62, 63
Red-fronted Serin	Serinus_pusillus	2	9	16	2	1.30	3.56	2.00	0.62	2.74	0.28	0.25	0.31	0.50	2	0.01	0.22	HPLC	3, 62, 65
European Serin	Serinus_serinus	2	9	16	2	1.30	3.56	2.00	0.62	2.74	0.28	0.25	0.31	0.50	2	0.01	0.22	HPLC	62, 64, 65
American Redstart	Setophaga_ruticilla	2	9	16	2	1.30	3.56	2.00	0.62	2.74	0.28	0.25	0.31	0.50	2	0.01	0.22	OTH	38
Saffron Finch	Sicalis_flaveola	1	1	0	0	0.00	0.00	0.00	0.00	.	.	.	0.00	0.00	1	.	1.00	OTH	38
Grey-tailed piha	Snowornis_subalaris	1	1	0	0	0.00	0.00	0.00	0.00	.	.	.	0.00	0.00	1			HPLC	103
Yellow-bellied Sapsucker	Sphyrapicus_varius	4	18	34	8	2.88	3.78	6.00	0.33	3.23	0.49	0.23	0.15	0.52	4	0.01	0.07	HPLC	68
Elegant Tern	Sterna_elegans	4	8	16	5	2.33	4.00	5.00	0.37	2.55	0.37	0.33	0.32	0.36	2	0.00	0.13	TLC	27
Peruvian meadowlark	Sturnella_bellicosa	2	6	5	2	1.29	1.67	2.00	0.00	1.67	0.35	0.20	0.27	0.50	2			HPLC	108
Eastern meadowlark	Sturnella_magna	1	1	0	0	0.00	0.00	0.00	0.00	.	.	.	0.00	0.00	1			HPLC	108
Red-breasted blackbird	Sturnella_militaris	4	16	32	8	2.91	4.00	6.00	0.37	3.39	0.50	0.26	0.18	0.47	4			HPLC	108
Western meadowlark	Sturnella_neglecta	1	1	0	0	0.00	0.00	0.00	0.00	.	.	.	0.00	0.00	1			HPLC	108
White-browed blackbird	Sturnella_superciliaris	4	16	32	8	2.91	4.00	6.00	0.37	3.39	0.50	0.26	0.18	0.47	4			HPLC	108
Zebra Finch	Taeniopygia_guttata	4	18	36	9	3.06	4.00	6.00	0.29	3.38	0.47	0.22	0.16	0.48	4	0.01	0.07	HPLC	41, 44
Golden Bush-robin	Tarsiger_chrysaeus	2	3	3	2	1.25	2.00	1.00	0.00	1.67	0.35	1.00	0.67	0.00	1	0.00	0.33	HPLC	63
Sulfur-breasted Bushrike	Telophorus_sulfureopectus	2	11	21	5	2.10	3.82	4.00	0.40	3.22	0.35	0.28	0.27	0.40	3	0.02	0.14	HPLC	63
Bokmakierie	Telophorus_zeylonus	1	4	6	2	1.14		1.00	0.46	2.67	0.20	0.33	0.83	0.00	1			HPLC	111
Capercaillie	Tetrao_urogallus	2	6	10	2	1.38	3.33	2.00	0.63	2.80	0.58	0.60	0.40	0.11	3	0.00	0.31	TLC	16
Wallcreeper	Tichodroma_muraria	1	5	10	2	1.38	4.00	2.00	0.75	2.80	0.33	0.67	0.60	0.11	2	0.00	0.44	HPLC	62
Black-and-gold Cotinga	Tijuca_atra	1	1	0	0	0.00	0.00	0.00	0.00	.	.	.	0.00	0.00	1			HPLC	103
Ecuadorian trogon	Trogon_mesurus	4	16	32	8	2.91	4.00	7.00	0.37	3.39	0.50	0.26	0.18	0.47	4			HPLC	111
Eurasian Blackbird	Turdus_merula	5	5	0	0	0.00	0.00	0.00	0.00	.	.	.	0.00	0.00	5	.	0.20	HPLC	19
Cassin's kingbird	Tyrannus_vociferans	2	4	2	2	1.33		2.00	0.00	1.67	0.71	0.67	0.33	0.00	2			HPLC	111
Long-tailed Rosefinch	Uragus_sibiricus	3	13	20	6	2.27	3.08	6.00	0.23	2.82	0.60	0.38	0.18	0.42	4	0.02	0.10	HPLC	62, 63, 67, 69
Nashville Warbler	Vermivora_ruficapilla	1	1	0	0	0.00	0.00	0.00	0.00	.	.	.	0.00	0.00	1	.	1.00	TLC	5
Virginia's Warbler	Vermivora_virginiae	1	1	0	0	0.00	0.00	0.00	0.00	.	.	.	0.00	0.00	1	.	1.00	TLC	5
I'iwi	Vestiaria_coccinea	4	16	31	8	3.09	3.88	7.00	0.37	3.08	0.45	0.19	0.17	0.51	4			HPLC	111
Yellow-headed blackbird	Xanthocephalus_xanthocephalus	1	1	0	0	0.00	0.00	0.00	0.00	.	.	.	0.00	0.00	1			HPLC	108, 109, 110
White-winged cotinga	Xipholena_atropurpurea	4	15	23	8	3.19	3.45	5.00	0.26	3.16	0.55	0.19	0.12	0.68	4			HPLC	103
White-tailed cotinga	Xipholena_lamellipennis	4	16	28	8	3.21	3.52	5.00	0.27	3.23	0.55	0.20	0.12	0.68	5			HPLC	103
Pompadour cotinga	Xipholena_punicea	4	16	29	8	3.38	3.39	8.00	0.25	3.09	0.54	0.18	0.11	0.66	5			HPLC	103,104
Japanese White-eye	Zosterops_japonicus	1	1	0	0	0.00	0.00	0.00	0.00	.	.	.	0.00	0.00	1	.	1.00	OTH	38

Method:

**HPLC**	High-performance liquid chromatography
**TLC**	Thin layer chromatography
**HPLC2**	HPLC and TLC combined
**HPLC3**	HPLC and Mass spectrometry
**OTH**	Mass spectrometry, others

Literature Sources for Appendix S2:

1 Andersson, S., Prager, M. & Johansson, E. I. A. Carotenoid content and reflectance of yellow and red nuptial plumages in widowbirds (Euplectes spp.). Functional Ecology 21, 272-281 (2007).

2 Arnold, K. E., Ramsay, S. L., Henderson, L. & Larcombe, S. D. Seasonal variation in diet quality: antioxidants, invertebrates and blue tits Cyanistes caeruleus. Biological Journal of the Linnean Society 99, 708-717 (2010).

3 Badyaev, A. V., Belloni, V., Kennedy, L. & Delaney, R. (unpubl. data).

4 Brockmann, H. & Völker, O. Der gelbe Federfarbstoff des Kanarienvogels [Serinus canaria canaria (L.)] und das Vorkommen von Carotinoiden bei Vögeln. Hoppe-Seyler's Zeitschrift für physiologische Chemie 224, 193-215 (1934).

5 Brush, A. H. & Johnson, N. K. The Evolution of Color Differences between Nashville and Virginia's Warblers. Condor 78, 412-414 (1976).

6 Brush, A. H. & Power, D. M. House finch pigmentation: carotenoid metabolism and the effect of diet. Auk 93, 725-739 (1976).

7 Brush, A. H. Pigmentation in the scarlet tanager, Piranga olivacea. Condor 69, 549-559 (1967).

8 Brush, A. H. & Allen, K. Astaxanthin in the Cedar Waxwing. Science 142, 47-48 (1963).

9 Butler, M. W. & McGraw, K. J. Relationships between dietary carotenoids, body tissue carotenoids, parasite burden, and health state in wild mallard (Anas platyrhynchos) ducklings. Arch Biochem Biophys 504, 154-160, doi:10.1016/j.abb.2010.07.003 (2010).

10 Chui, C. K. S., McGraw, K. J. & Doucet, S. M. Carotenoid-based plumage coloration in golden-crowned kinglets Regulus satrapa: pigment characterization and relationships with migratory timing and condition. Journal of Avian Biology 42, 309-322 (2011).

11 Cohen, A. A., McGraw, K. J. & Robinson, W. D. Serum antioxidant levels in wild birds vary in relation to diet, season, life history strategy, and species. Oecologia 161, 673-683, doi:10.1007/s00442-009-1423-9 (2009).

12 Czeczuga, B. Carotenoids in the skin of certain species of birds. Comparative Biochemistry and Physiology Part B: Comparative Biochemistry 62, 107-109 (1979).

13 del Val, E. et al. The liver but not the skin is the site for conversion of a red carotenoid in a passerine bird. Naturwissenschaften 96, 797-801, doi:10.1007/s00114-009-0534-9 (2009).

14 Deviche, P., McGraw, K. J. & Underwood, J. Season-, sex-, and age-specific accumulation of plasma carotenoid pigments in free-ranging white-winged crossbills Loxia leucoptera. Journal of Avian Biology 39, 283-292 (2008).

15 Eeva, T., Sillanpää, S. & Salminen, J. P. The effects of diet quality and quantity on plumage colour and growth of great tit Parus major nestlings: a food manipulation experiment along a pollution gradient. Journal of Avian Biology 40, 491-499 (2009).

16 Egeland, E. S., Parker, H. & Liaaenjensen, S. Carotenoids in combs of Capercaillie (Tetrao urogallus) fed defined diets. Poultry Science 72, 747-751 (1993).

17 Ewen, J. G. et al. Carotenoids, colour and conservation in an endangered passerine, the hihi or stitchbird (Notiomystis cincta). Anim Conserv 9, 229-235 (2006).

18 Ewen, J. G., Thorogood, R., Karadas, F., Pappas, A. C. & Surai, P. F. Influences of carotenoid supplementation on the integrated antioxidant system of a free living endangered passerine, the hihi (Notiomystis cincta). Comparative Biochemistry and Physiology - Part A: Molecular & Integrative Physiology 143, 149-154 (2006).

19 Faivre, B., Gregoire, A., Preault, M., Cezilly, F. & Sorci, G. Immune activation rapidly mirrored in a secondary sexual trait. Science 300, 103 (2003).

20 Fox, D. L. & Hopkins, T. S. Comparative metabolic fractionation of carotenoids in three flamingo species. Comparative Biochemistry and Physiology 17, 841-856 (1966).

21 Fox, D. L. Carotenoids of the scarlet ibis. Comparative Biochemistry and Physiology 5, 31-43 (1962).

22 Fox, D. L., Smith, V. E. & Wolfson, A. A. Carotenoid selectivity in blood and feathers of lesser (African), chilean and greater (European) flamingos. Comparative Biochemistry and Physiology 23, 225-232 (1967).

23 Fox, D. L., Hopkins, T. S. & Zilversmit, D. B. Blood carotenoids of the roseate spoonbill. Comparative Biochemistry and Physiology 14, 641-649 (1965).

24 Fox, D. L., Wolfson, A. A. & McBeth, J. W. Metabolism of b-carotene in the American flamingo, Phoenicopterus ruber. Comparative Biochemistry and Physiology 29, 1223-1229 (1969).

25 Hõrak, P., Surai, P. F., Ots, I. & Møller, A. P. Fat soluble antioxidants in brood-rearing great tits Parus major: relations to health and appearance. Journal of Avian Biology 35, 63-70 (2004).

26 Hudon, J. & Brush, A. H. Probably dietary basis of a color variant of the cedar waxwing. Journal of Field Ornithology 60, 361-368 (1989).

27 Hudon, J. & Brush, A. H. Carotenoids produce flush in the elegant tern plumage. Condor 92, 798-801 (1990).

28 Hudon, J. Unusual carotenoid use by western tanager (Piranga ludoviviana) and its evolutionary implications. Canadian Journal of Zoology 69, 2311-2320 (1991).

29 Hudon, J., Capparella, A. P. & Brush, A. H. Plumage pigment differences in manakins of the Pipra erythrocephala superspecies Auk 106, 34-41 (1989).

30 Hudon, J., Ouellet, H., Bénito-Espinal, É. & Brush, A. H. Characterization of an Orange Variant of the Bananaquit (Coereba flaveola) on La Désirade, Guadeloupe, French West Indies. Auk 113, 715-718 (1996).

31 Hudon, J., Anciaes, M., Bertacche, V. & Stradi, R. Plumage carotenoids of the Pin-tailed Manakin (Ilicura militaris): evidence for the endogenous production of rhodoxanthin from a colour variant. Comparative biochemistry and physiology. Part B, Biochemistry & molecular biology 147, 402-411, doi:10.1016/j.cbpb.2007.02.004 (2007).

32 Inouye, C. Y., Hill, G. E., Stradi, R. D., Montgomerie, R. & Bosque, C. Carotenoid pigments in male house finch plumage in relation to age, subspecies, and ornamental coloration. Auk 118, 900-915 (2001).

33 Isaksson, C., Ornborg, J., Prager, M. & Andersson, S. Sex and age differences in reflectance and biochemistry of carotenoid-based colour variation in the great tit Parus major. Biological Journal of the Linnean Society 95, 758-765 (2008).

34 Isaksson, C., Sturve, J., Almroth, B. C. & Andersson, S. The impact of urban environment on oxidative damage (TBARS) and antioxidant systems in lungs and liver of great tits, Parus major. Environ Res 109, 46-50 (2009).

35 Johnson, N. K. & Brush, A. H. Analysis of Polymorphism in the Sooty-Capped Bush Tanager. Systematic Zoology 21, 245-262 (1972).

36 Juola, F. A., McGraw, K. J. & Dearborn, D. C. Carotenoids and throat pouch coloration in the great frigatebird (Fregata minor). Comparative Biochemistry and Physiology Part B: Biochemistry and Molecular Biology 149, 370-377 (2008).

37 Mays Jr, H. L. et al. Sexual dichromatism in the yellow-breasted chat Icteria virens: spectrophotometric analysis and biochemical basis. Journal of Avian Biology 35, 125-134 (2004).

38 McGraw, K. J. in Bird Coloration. I. Mechanisms and Measurements (eds G.E. Hill & K. J. McGraw) 177-242 (Harvard University Press, 2006).
39 McGraw, K. J. & Hardy, L. S. Astaxanthin is responsible for the pink plumage flush in Franklin's and Ring-billed gulls. Journal of Field Ornithology 77, 29-33 (2006).
40 McGraw, K. J. & Schuetz, J. G. The evolution of carotenoid coloration in estrildid finches: a biochemical analysis Comp. Biochem. Physiol. B 139, 45-51 (2004).
41 McGraw, Kevin J. & Toomey, Matthew B. Carotenoid Accumulation in the Tissues of Zebra Finches: Predictors of Integumentary Pigmentation and Implications for Carotenoid Allocation Strategies. Physiological and Biochemical Zoology 83, 97-109, doi:doi:10.1086/648396 (2010).
42 McGraw, K. J., Nolan, P. M. & Crino, O. L. Carotenoid accumulation strategies for becoming a colourful House Finch: analyses of plasma and liver pigments in wild moulting birds. Functional Ecology 20, 678-688 (2006).
43 McGraw, K. J., Hill, G. E., Stradi, R. & Parker, R. S. The Influence of Carotenoid Acquisition and Utilization on the Maintenance of Species-Typical Plumage Pigmentation in Male American Goldfinches (Carduelis tristis) and Northern Cardinals (Cardinalis cardinalis). Physiological and Biochemical Zoology 74, 843-852, doi:doi:10.1086/323797 (2001).
44 McGraw, K. J., Adkins-Regan, E. & Parker, R. S. Anhydrolutein in the zebra finch: a new, metabolically derived carotenoid in birds. Comparative Biochemistry and Physiology Part B: Biochemistry and Molecular Biology 132, 811-818 (2002).
45 McGraw, K. J., Beebee, M. D., Hill, G. E. & Parker, R. S. Lutein-based plumage coloration in songbirds is a consequence of selective pigment incorporation into feathers. Comparative Biochemistry and Physiology Part B: Biochemistry and Molecular Biology 135, 689-696 (2003).
46 McGraw, K. J., Hill, G. E. & Parker, R. S. Carotenoid pigments in a mutant cardinal: Implications for the genetic and enzymatic control mechanisms of carotenoid metabolism in birds. Condor 105, 587-592 (2003).
47 McGraw, K. J., Wakamatsu, K., Clark, A. B. & Yasukawa, K. Red-winged blackbirds Agelaius phoeniceus use carotenoid and melanin pigments to color their epaulets. Journal of Avian Biology 35, 543-550 (2004).
48 McGraw, K. J., Hill, G. E. & Parker, R. S. The physiological costs of being colourful: nutritional control of carotenoid utilization in the American goldfinch, Carduelis tristis. Animal Behaviour 69, 653-660, doi:10.1016/j.anbehav.2004.05.018 (2005).
49 Negro, J. J. & Garrido-Fernández, J. Astaxanthin is the major carotenoid in tissues of white storks (Ciconia ciconia) feeding on introduced crayfish (Procambarus clarkii). Comparative Biochemistry and Physiology Part B: Biochemistry and Molecular Biology 126, 347-352 (2000).
50 Negro, J. J., Tella, J. L., Hiraldo, F., Bortolotti, G. R. & Prieto, P. Sex- and age-related variation in plasma carotenoids despite a constant diet in the red-legged partridge (Alectoris rufa). Ardea 89, 275-279 (2001).
51 Negro, J. J. et al. Coprophagy: An unusual source of essential carotenoids. Nature 416, 807-808 (2002).
52 Pérez, C., Lores, M. & Velando, A. Availability of nonpigmentary antioxidant affects red coloration in gulls. Behavioral Ecology 19, 967-973, doi:10.1093/beheco/arn053 (2008).
53 Peters, A., Delhey, K., Denk, A. G. & Kempenaers, B. Tarde-offs between immune investment and sexual signaling in male mallards. American Naturalist 164, 51-59 (2004).
54 Peters, A., Delhey, K., Andersson, S., Van Noordwijk, H. & Förschler, M. I. Condition-dependence of multiple carotenoid-based plumage traits: an experimental study. Functional Ecology 22, 831-839 (2008).
55 Prager, M., Johansson, E. I. & Andersson, S. Differential ability of carotenoid C4-oxygenation in yellow and red bishop species (Euplectes spp.). Comparative biochemistry and physiology. Part B, Biochemistry & molecular biology 154, 373-380, doi:10.1016/j.cbpb.2009.06.015 (2009).
56 Quesada, J. & Senar, J. C. Comparing plumage colour measurements obtained directly from live birds and from collected feathers: the case of the great tit Parus major. Journal of Avian Biology 37, 609-616 (2006).
57 Rowe, M. & McGraw, K. J. Carotenoids in the Seminal Fluid of Wild Birds: Interspecific Variation in Fairy-Wrens. The Condor 110, 694-700, doi:10.1525/cond.2008.8604 (2008).
58 Ryan, P. G., Moloney, C. L. & Hudon, J. Color variation and hybridization among Nesospiza Buntings on inaccessible islands, Tristan da Cunha. Auk 111, 314-327 (1994).
59 Saino, N., Bertacche, V., Bonisoli-Alquati, A., Romano, M. & Rubolini, D. Phenotypic Correlates of Yolk and Plasma Carotenoid Concentration in Yellow-Legged Gull Chicks. Physiological and Biochemical Zoology 81, 211-225, doi:doi:10.1086/527454 (2008).
60 Saks, L., McGraw, K. & Hõrak, P. How feather colour reflects its carotenoid content. Functional Ecology 17, 555-561 (2003).
61 Sillanpää, S., Salminen, J.-P. & Eeva, T. Breeding success and lutein availability in great tit (Parus major). Acta Oecologica 35, 805-810 (2009).
62 Stradi, R. The Colour of Flight. (Solei Gruppos Editoriale Informatico, 1998).
63 Stradi, R. in Colori in volo - il piumaggio degli uccelli (eds l. Brambilla, G. Canali, Mannucci E., & et al.) 117-146 (Università degli Studi di Milano 1999).
64 Stradi, R., Celentano, G. & Nava, D. Separation and identification of carotenoids in bird's plumage by high-performance liquid chromatography-diode-array detection. Journal of Chromatography B: Biomedical Sciences and Applications 670, 337-348 (1995).
65 Stradi, R., Celentano, G., Rossi, E., Rovati, G. & Pastore, M. Carotenoids in bird plumage: I. The carotenoid pattern in a series of Palearctic Carduelinae Comparative Biochemestry Physiology Part B: Comparative Biochemistry and Physiology 110, 131 -143 (1995).
66 Stradi, R., Rossi, E., Celentano, G. & Bellardi, B. Carotenoids in bird plumage: the pattern in three Loxia species and in Pinicola enucleator. Comparative Biochemistry and Physiology Part B: Biochemistry and Molecular Biology 113, 427-432 (1996).
67 Stradi, R., Celentano, G., Boles, M. & Mercato, F. Carotenoids in Bird Plumage: The Pattern in a Series of Red-Pigmented Carduelinae. Comparative Biochemistry and Physiology Part B: Biochemistry and Molecular Biology 117, 85-91 (1997).
68 Stradi, R., Hudon, J., Celentano, G. & Pini, E. Carotenoids in bird plumage: the complement of yellow and red pigments in true woodpeckers (Picinae). Comparative Biochemistry and Physiology Part B: Biochemistry and Molecular Biology 120, 223-230 (1998).
69 Stradi, R., Pini, E. & Celentano, G. Carotenoids in bird plumage: the complement of red pigments in the plumage of wild and captive bullfinch (Pyrrhula pyrrhula). Comparative Biochemistry and Physiology Part B 128, 529-535 (2001).
70 Toomey, M. B. & McGraw, K. J. Seasonal, sexual, and quality related variation in retinal carotenoid accumulation in the house finch (Carpodacus mexicanus). Functional Ecology 23, 321-329 (2009).

71 Toomey, M. B. & McGraw, K. J. The effects of dietary carotenoid intake on carotenoid accumulation in the retina of a wild bird, the house finch (Carpodacus mexicanus). Arch Biochem Biophys 504, 161-168 (2010).
72 Tyczkowski, J. K., Yagen, B. & Hamilton, P. B. Metabolism of canthaxanthin, a red diketocarotenoid, by chickens. Poultry Science 67, 787-793 (1988).
100. García-de Blas, E., R. Mateo, J. Viñuela, L. Pérez-Rodríguez, and C. Alonso-Alvarez. 2013. Free and esterified carotenoids in ornaments of an avian species: the relationship to color expression and sources of variability. Physiological and Biochemical Zoology 86:483-498.
101. García-de Blas, E., R. Mateo, F. Guzmán Bernardo, R. Rodríguez Martín-Doimeadios, and C. Alonso-Alvarez. 2014. Astaxanthin and papilioerythrinone in the skin of birds: a chromatic convergence of two metabolic routes with different precursors? Naturwissenschaften 101:407-416.
102. Mendes-Pinto, M. M., A. M. LaFountain, M. C. Stoddard, R. O. Prum, H. A. Frank, and B. Robert. 2012. Variation in carotenoid–protein interaction in bird feathers produces novel plumage coloration. Journal of The Royal Society Interface 9:3338-3350.
103. Prum, R. O., A. M. LaFountain, J. Berro, M. C. Stoddard, and H. A. Frank. 2012. Molecular diversity, metabolic transformation, and evolution of carotenoid feather pigments in cotingas (Aves: Cotingidae). J Comp Physiol B 182:1095-1116.
104. LaFountain, A. M., S. Kaligotla, S. Cawley, K. M. Riedl, S. J. Schwartz, H. A. Frank, and R. O. Prum. 2010. Novel methoxy-carotenoids from the burgundy-colored plumage of the Pompadour Cotinga Xipholena punicea. Archives of Biochemistry and Biophysics 504:142-153.
105. Hudon, J., A. Storni, E. Pini, M. Anciães, and R. Stradi. 2012. Rhodoxanthin as a Characteristic Keto-Carotenoid of Manakins (Pipridae). The Auk 129:491-499.
106. Prum, R., A. LaFountain, C. Berg, M. Tauber, and H. Frank. 2014. Mechanism of carotenoid coloration in the brightly colored plumages of broadbills (Eurylaimidae). J Comp Physiol B 184:651-672.
107. Friedman, N. R., K. J. McGraw, and K. E. Omland. 2014. History and mechanisms of carotenoid plumage evolution in the New World orioles (Icterus). Comparative Biochemistry and Physiology Part B: Biochemistry and Molecular Biology 172–173:1-8.
108. Friedman, N. R., K. J. McGraw, and K. E. Omland. 2014. Evolution of carotenoid pigmentation in Caciques and Meadowlarks (Icteridae): repeated gains of red plumage coloration by carotenoid C4-oxygenation. Evolution 68:791-801.
109. Newbrey, J. L., W. L. Reed, S. P. Foster, and G. L. Zander. 2008. Laying-Sequence Variation in Yolk Carotenoid Concentrations in Eggs of Yellow-Headed Blackbirds (Xanthocephalus xanthocephalus). The Auk 125:124-130.
110. Newbrey, J. L. and W. L. Reed. 2009. Growth of yellow-headed blackbird Xanthocephalus xanthocephalus nestlings in relation to maternal body condition, egg mass, and yolk carotenoids concentrations. Journal of Avian Biology 40:419-429.
111. Thomas, D. B., K. J. McGraw, H. F. James, and O. Madden. 2014. Non-destructive descriptions of carotenoids in feathers using Raman spectroscopy. Analytical Methods 6:1301-1308.
112. LaFountain, A. M., H. A. Frank, and R. O. Prum. 2013. Carotenoids from the crimson and maroon plumages of Old World orioles (Oriolidae). Archives of Biochemistry and Biophysics 539:126-132.
113. Berg, C. J., A. M. LaFountain, R. O. Prum, H. A. Frank, and M. J. Tauber. 2013. Vibrational and electronic spectroscopy of the retro-carotenoid rhodoxanthin in avian plumage, solid-state films, and solution. Archives of Biochemistry and Biophysics 539:142-155.

Appendix S3. Module assignments in the avian subset of the global carotenoid metabolic network

CAROTENOID	Module Assignment
lutein	1
(3R, 3'R) zeaxanthin	5
β-carotene	6
β-cryptoxanthin	6
anhydrolutein	1
7,8-dihydrolutein	1
9-Z-7,8-dihydrolutein	1
canary xanthophyll A	1
canary xanthophyll B	1
α-doradexanthin	3
(3S,4R,3'R,6'R) 4-hydroxylutein	3
fritschiellaxanthin	3
papilioerythrinone	3
3'-dehdyrolutein	1
piprixanthin	4
rhodoxanthin	4
7,8,7',8'-tetrahydrozeaxanthin	5
7,8-dihydrozeaxanthin	5
idoxanthin	5
fucoxanthin	8
7,8 dihydro β-cryptoxanthin	6
4-hydroxyzeaxanthin	5
adonixanthin	5
13 cis-(3S,3'S) astaxanthin	5
echineone	6
3'-hydroxyechinenone	6
canthaxanthin	6
adonirubin	6
4-hydroxy-echinenone	6
isozeaxanthin	6
β-isocryptoxanthin	6
α-carotene	2
α-isocryptoxanthin	2
phoenicopterone	2
α-cryptoxanthin	2
rubixanthin	9
4-oxo-rubixanthin	9
gazaniaxanthin	10
4-oxo-gazaniaxanthin	10
(3S,4R,3'S,6'R) 4-hydroxylutein	3
cis lutein	1
resonance stabilized form	4
xipholenin	3
2,3-didehydro-xipholenin	3
rupicolin	5
3'-hydroxy-3-methoxy-canthaxanthin	7
pompadourin	7
2,3-didehydro-pompadourin	7
cotingin	7
brittonxanthin	6
cymbirhynchin	3
7,8-dihydro-3'-dehydrolutein	1
4-hydroxy-canary xanthophyll A	1

Module numbers correspond to the paritioned regions in Fig. 2.
Compounds that were not found in the plumage and/or integument of birds were not included.
See Methods for details on how modules were assigned.
